# The *Plasmodium falciparum* NCR1 transporter is an antimalarial target that exports cholesterol from the parasite’s plasma membrane

**DOI:** 10.1126/sciadv.adq6651

**Published:** 2024-12-18

**Authors:** Zhemin Zhang, Meinan Lyu, Xu Han, Sepalika Bandara, Meng Cui, Eva S. Istvan, Xinran Geng, Marios L. Tringides, William D. Gregor, Masaru Miyagi, Jenna Oberstaller, John H. Adams, Youwei Zhang, Marvin T. Nieman, Johannes von Lintig, Daniel E. Goldberg, Edward W. Yu

**Affiliations:** ^1^ Department of Pharmacology, Case Western Reserve University School of Medicine, Cleveland, OH 44106, USA.; ^2^ Department of Pharmaceutical Sciences, Northeastern University School of Pharmacy, Boston, MA 02115, USA.; ^3^ Division of Infectious Diseases, Department of Medicine, Washington University School of Medicine, St. Louis, MO 63110, USA.; ^4^ Department of Molecular Microbiology, Washington University School of Medicine, St. Louis, MO 63110, USA.; ^5^ Center for Global Health and Infectious Diseases, Department of Global Health, University of South Florida, 3720 Spectrum Boulevard, Suite 404, Tampa, FL 33612, USA.

## Abstract

Malaria, a devastating parasitic infection, is the leading cause of death in many developing countries. Unfortunately, the most deadliest causative agent of malaria, *Plasmodium falciparum*, has developed resistance to nearly all currently available antimalarial drugs. The *P. falciparum* Niemann-Pick type C1–related (PfNCR1) transporter has been identified as a druggable target, but its structure and detailed molecular mechanism are not yet available. Here, we present three structures of PfNCR1 with and without the functional inhibitor MMV009108 at resolutions between 2.98 and 3.81 Å using single-particle cryo–electron microscopy (cryo-EM), suggesting that PfNCR1 binds cholesterol and forms a cholesterol transport tunnel to modulate the composition of the parasite plasma membrane. Cholesterol efflux assays show that PfNCR1 is an exporter capable of extruding cholesterol from the membrane. Additionally, the inhibition mechanism of MMV009108 appears to be due to a direct blockage of PfNCR1, preventing this transporter from shuttling cholesterol.

## Teaser

PfNCR1 exports cholesterol to regulate the composition of the parasite plasma membrane.

## INTRODUCTION

Malaria remains a persistent global health problem, with the most vulnerable groups being young children and pregnant women. *Plasmodium falciparum* is the most prevalent and deadliest causative agent of malaria. In 2021, this parasite was responsible for causing the disease in approximately 247 million people and claiming 619,000 lives, with 96% of the mortalities located in Africa (*1*). Unfortunately, *P. falciparum* has developed resistance to nearly all currently available antimalarial drugs, including sulfadoxine, pyrimethamine, mefloquine, halofantrine, and quinine (*2*). Resistance to highly effective artemisinin-combination therapies first emerged in parts of Southeast Asia (*3*) and later observed in Africa. The impact of multidrug-resistant malaria is an enormous global health threat, and the identification of classes of antimalarials and druggable targets should be a global health priority.

It is known that the malarial parasite *P. falciparum* relies on cholesterol (Chl) for growth and invasion. However, *P. falciparum* is not capable of synthesizing this essential molecule de novo and must acquire Chl from the host for survival and development (*4*). *P. falciparum* expresses several proteins that likely play important roles in uptake, binding, and intracellular trafficking of Chl (*5*). It has been observed that several human host cells, including liver and red blood cells, are rich in Chl, and these cells are particularly favorable for malarial parasites to reside and grow (*5*).

Although Chl is a prerequisite to the invasion and growth of *P. falciparum* in the host, it appears that this can be a double-edged sword, as the parasite has to carefully control and maintain a certain level of Chl for successful infection. It has been observed that artificially reducing the Chl concentration in erythrocytes leads to less infected cells (*6*–*8*). However, artificially increasing the Chl level to high concentrations in erythrocytes results in a decrease in the efficiency of the parasite’s invasion (*6*, *9*). After invasion, the parasite must maintain the level of Chl in the host’s plasma membrane above a certain threshold. It has also been observed that the removal of Chl from the erythrocyte’s plasma membrane leads to a rapid extrusion of the parasite from the infected erythrocyte (*4*, *8*). Therefore, *P. falciparum* must develop an effective mechanism to regulate Chl levels and membrane homeostasis.

*P. falciparum* has a large number of membrane proteins with unidentified function. One such membrane protein is the *P. falciparum* Niemann-Pick type C1–related protein (PfNCR1), which is encoded by the gene PF3D7_0107500 (*10*). PfNCR1 is homologous to the human Niemann-Pick C1 (hNPC1) transporter (*11*). These two-membrane protein shares 23% identity and 44% similarity in protein sequence. Although the function of PfNCR1 is not completely clear, it has been proposed to be engaged in Chl trafficking (*10*). This gene is resistant to transposon mutagenesis (*12*), and a genetic knockdown study provides further evidence that *pfncr1* is an essential gene, critical for blood-stage parasite replication (*10*). Further, *pfncr1* knockdown causes the parasite to become hypersensitive to saponin, a pore-forming amphipathic glycoside with an affinity for free Chl. PfNCR1 localizes to the parasite’s plasma membrane (PPM) at zones of contact with the parasitophorous vacuolar membrane that surrounds the parasite (*13*). Hence, inhibition via genetic knockdown of the PfNCR1 transporter leads to the accumulation of Chl on the PPM, indicating that PfNCR1 may be responsible for removing Chl from the parasite.

PfNCR1 is an attractive druggable target, as it is necessary for maintaining proper membrane lipid composition at the blood stage (*10*). To initiate our research efforts to uncover antimalarial drug targets and their mechanism of action to facilitate the development of antimalarial therapeutics, we decided to elucidate the structural basis of the PfNCR1 membrane protein. Our work detailed here provides molecular insights into the transport mechanism of Chl mediated by the PfNCR1 membrane protein to regulate membrane homeostasis. We present structures of PfNCR1 bound with Chl using single-particle cryo–electron microscopy (cryo-EM) to resolutions of 3.11 and 3.81 Å. We also report the 2.98-Å cryo-EM structure of PfNCR1 bound with MMV009108, a potential PfNCR1 inhibitor that heightens the sensitivity of *P. falciparum* to saponin. The structural information suggests that PfNCR1 contains two different binding sites for Chl and that this membrane protein forms a tunnel to shuttle Chl between these two binding sites. It also enabled us to determine that MMV009108 creates a blockage within PfNCR1, forbidding it to shuttle Chl. Combined, cryo-EM, Chl efflux assays, and molecular dynamics (MD) and targeted MD (TMD) simulations demonstrate that the PfNCR1 protein is capable of exporting Chls from the PPM to the parasitophorous vacuole (PV) to facilitate membrane homeostasis.

## RESULTS

### Structural determination of the PfNCR1 transporter

To elucidate the structural information of PfNCR1, we cloned the gene *pfncr1* (PF3D7_0107500), which encodes the full-length *P. falciparum* PfNCR1 transporter of 1470 amino acids, into the pcDNA3.1-N-DYK expression plasmid. PfNCR1 was expressed in human embryonic kidney (HEK) 293 cells and purified using a strep-tactin affinity column. We further purified PfNCR1 using a Superose 6 column and collected single-particle cryo-EM data of this membrane protein. Extensive classification of these single-particle images indicated that there were two distinct populations of PfNCR1 with different conformations coexisting in the single protein sample (fig. S1). Several iterative rounds of classifications allowed us to sort the images based on these two distinct conformations. Three-dimensional (3D) reconstitutions of the two PfNCR1 transporter classes led to cryo-EM maps at nominal resolutions of 3.11 Å (PfNCR1-I) and 3.81 Å (PfNCR1-II) (table S1 and fig. S1), which enabled us to build two structural models of the PfNCR1 membrane protein to these resolutions. Each final structural model of the PfNCR1 transporter includes residues 3–180, 293–673, 719–736, 1046–1151, and 1169–1470.

#### Structure of PfNCR1-I

**Fig. 1.**
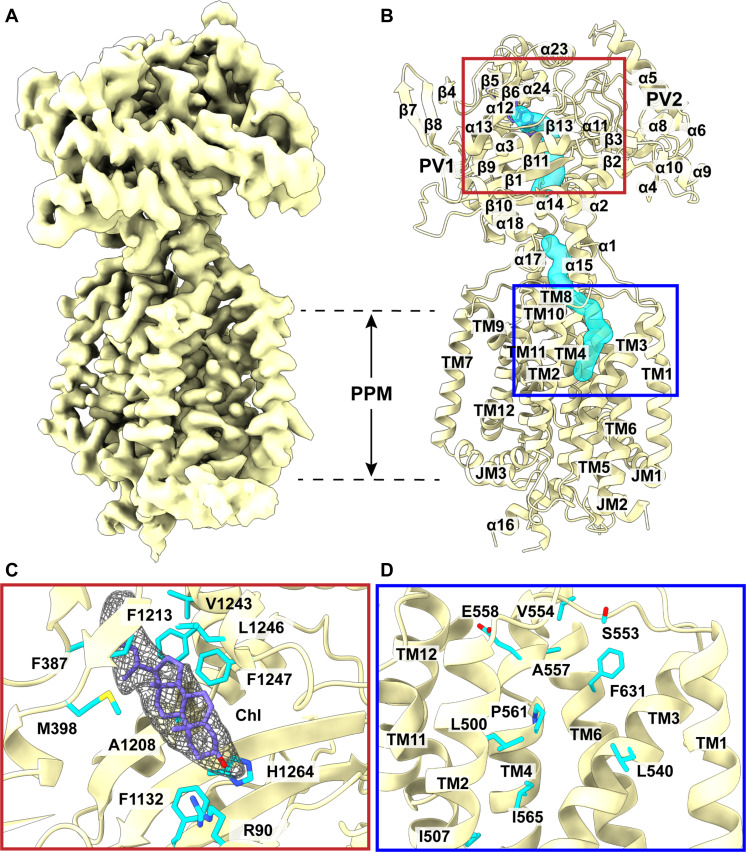
Structure of PfNCR1-I. (**A**) Cryo-EM map of PfNCR1-I at a resolution of 3.11 Å. (**B**) Side view of the ribbon diagram of PfNCR1-I viewed in the membrane plane. The structure of PfNCR1-I indicates that this transporter contains the PPM and PV domains. The PPM domain consists of 12 TMs, whereas the PV domain is composed of subdomains PV1 and PV2. The PfNCR1 transporter forms a channel (cyan) spanning the PV domain and down to the outer leaflet of the PPM domain. This channel is broken into two halves. It appears that residues 1105–1108, which form a short α17, block and split this tunnel into two portions. (**C**) Chl binding site. Cryo-EM density of bound Chl is in gray meshes. The bound Chl molecule is in slate sticks. The binding residues R90, F387, M398, F1132, A1208, F1213, V1243, L1246, F1247, and H1264 are in cyan sticks. (**D**) Sterol-sensitive domain (SSD). Residues L500, I507, L540, S553, V554, A557, E558, P561, I565, and F631 (cyan sticks) are found to surround the cavity formed at the SSD. These residues are expected to be important for substrate binding.

Our cryo-EM structure indicates that PfNCR1 is monomeric in form with overall dimensions of 110 Å × 75 Å × 55 Å. This transporter consists of a large membrane-spanning domain formed by 12 transmembrane helices (TM1 to TM12) and a large extension formed by two hydrophilic loops (loops 1 and 2), consistent with the signature of the resistance–nodulation–cell division (RND) superfamily of proteins (Fig. 1, A and B) (*14*). It has been reported that the transmembrane domain of PfNCR1 resides at the PPM, while the two soluble loops protrude into and localize to the PV (*10*). These two large loops are located between TM1 and TM2, and between TM7 and TM8, where they contribute to subdomains PV1 and PV2 in the parasitophorous vacuolar space. Therefore, our cryo-EM structure depicts that the PfNCR1 transporter contains two domains: the transmembrane PPM domain consisting of 12 TMs (TM1 to TM12) and the soluble PV domain composed of subdomains (PV1 and PV2).

The N-terminal and C-terminal halves of PfNCR1 are assembled in a twofold pseudo-symmetrical fashion. These two halves can be superimposed; however, the root mean square deviation (RMSD) of this superimposition is very high (15.9 Å for 386 Cα atoms), suggesting that the structures of these two halves are very different from each other. PV1 is composed of 12 α helices and 10 β strands (Fig. 1B and fig. S2). Most PV1 amino acids emerge from loop 1. However, residues 1105–1125 of loop 2 also contribute to form helices α17 and α18 of PV1. PV2 constitutes 12 α helices and five β strands (Fig. 1B and fig. S2). Similar to PV1, the PV2 amino acids mainly arise from loop 2, but residues 63–84, 142–161, and 295–313 of loop 1 participate in the formation of helices α1, α2, α5, α6, and α7 of this subdomain. The crossover of these two loops allows for the two subdomains to be spatially adjoined within the PV. Four flexible linkers, constituted by residues 85–88, 128–141, 300–335, and 1126–1130, are responsible for connecting these two subdomains together. Within the transmembrane domain of PfNCR1, two juxtamembrane helices (JM1 and JM2), approximately parallel to the PPM, are located at the N terminus and between TM6 and TM7, respectively. These two JM helices may help strengthen the anchoring of this membrane protein in the PPM. The TMs are PPM embedded, but TM8 is substantially longer and protrudes into the PV region. TM8 also directly tethers PV2 and forms part of the PV domain structure. The 2D structural topology of PfNCR1 and its secondary structural elements, including TMs, JMs, α helices, and β strands, designated numerically from the N to C termini are shown in fig. S2.

A cleft is formed between subdomains PV1 and PV2. This cleft opens the top portion of the PV domain. A large cavity is also created at the gap between PV1 and PV2. The volume of this cavity is quite substantial and measured to be 6156 Å^3^. Potentially, this cavity can form a substrate-binding site to accommodate PfNCR1 substrates. In addition to this PV domain cavity, PfNCR1 also has a large cavity at the PPM domain. This cavity is located at the outer leaflet of the lipid bilayer and surrounded by TM2, TM3, and TM4, which create an internal space of 345 Å^3^. In view of the structure, the architecture of these TMs resembles the sterol-sensitive domain (SSD) found in both the human NPC1 (hNPC1) (*15*, *16*) and PTCH1 (hPTCH1) (*17*–*19*) membrane proteins. We used the DALI database (http://ekhidna2.biocenter.helsinki.fi/dali/) to search for membrane proteins that are homologous to PfNCR1. The program suggests that TM2 to TM4 of PfNCR1 constitute a cavity, mimicking the SSD that may be able to bind sterols and Chls (fig. S3).

The structure indicates that PfNCR1 constitutes a tunnel-like feature starting from the outer leaflet of the PPM to the vacuolar cleft of the PV. This tunnel spans the two large internal cavities at the PPM and PV domains of the transporter (Fig. 1B). Potentially, this tunnel may allow for the transport of substrates across these two large cavities. However, this tunnel is broken into two halves, suggesting that the structure of PfNCR1-I may depict a conformational state with a closed form of this tunnel. The constriction site is created by two flexible loops from each side of the tunnel, where residues L482, Y1105, L1301, and Y1305 are responsible for closing this tunnel (fig. S4). Residues lining the wall of this tunnel, including F54, I63, L66, F92, M398, F477, L482, V486, I489, I492, L500, L501, V504, F536, F539, L540, P555, P556, A557, P561, F1109, F1132, A1208, L1246, F1247, L1301, F1305, and F1436, are found to be hydrophobic. Presumably, this tunnel could help facilitate the transport of hydrophobic substrates.

Unexpectedly, a large extra EM density was found in the structure of PfNCR1. This extra density is observed within the large space between PV1 and PV2, near the ceiling of the PV domain. The shape of this EM density resembles a large, elongated sterol molecule (Fig. 1C). To identify this fortuitous ligand, we used gas chromatography coupled with mass spectrometry (GC-MS). GC-MS indicates that this ligand is cholest-5-en-3β-ol or Chl (fig. S5). Within 4.5 Å of the bound Chl molecule, there are at least 10 amino acids involved in the binding, R90, F387, M398, F1132, A1208, F1213, V1243, L1246, F1247, and H1264 (Fig. 1C). Most of the residues that form the Chl binding site are hydrophobic in nature. Likewise, the cavity formed by the SSD of PfNCR1-I is surrounded by 10 residues, including L500, I507, L540, S553, V554, A557, E558, P561, I565, and F631, with the majority of them hydrophobic (Fig. 1D).

Posttranslational oligosaccharide modifications have been found to play a critical role in localization, solubility, and stability of many eukaryotic proteins. In the PfNCR1 structure, two extra cryo-EM densities are observed to directly connect to residues N165 and N294, indicating that these two asparagines may form glycosylation sites. On the basis of the cryo-EM densities, each asparagine is connected to an *N*-acetylglucosamine (NAG) moiety (fig. S6). This structural feature is in good agreement with our liquid chromatography coupled with tandem mass spectrometry (LC-MS/MS) analysis, indicating that residues N165 and N294 are glycosylated (fig. S7). It should be noted that we expressed and purified PfNCR1 from HEK293 cells, which may introduce glycosylations different from when the protein is expressed in *Plasmodium* parasites.

#### Structure of PfNCR1-II

**Fig. 2.**
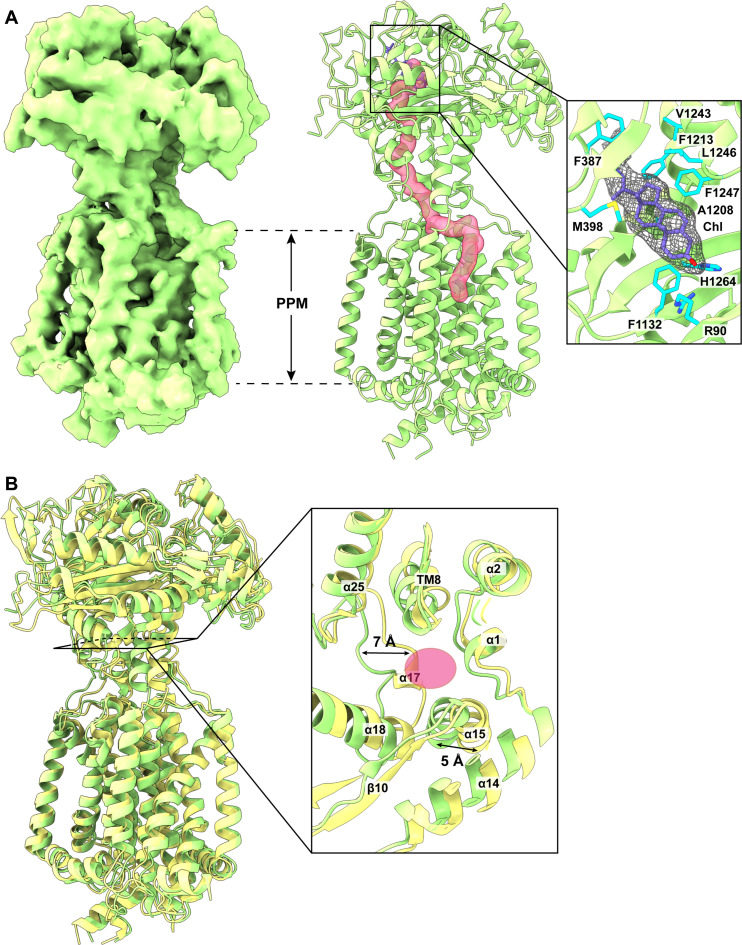
Structure of PfNCR1-II. (**A**) Cryo-EM map and side view of the ribbon diagram of PfNCR1-II viewed in the membrane plane at a resolution of 3.81 Å. Like PfNCR1-I, the structure of PfNCR1-II contains the PPM and PV domains. A continuous channel (pink) spanning the outer leaflet of the PPM domain and up to the PM domain. This tunnel is open in conformation, suggesting that the structure of PfNCR1-II represents the open-tunnel form of this transporter. A bound Chl molecule is found to occupy within the Chl binding site between subdomains PV1 and PV2 of PfNCR1-II. This bound Chl is represented by slate sticks. The cryo-EM density of bound Chl is in gray meshes. Residues R90, F387, M398, F1132, A1208, F1213, V1243, L1246, F1247, and H1264, responsible for anchoring Chl, are in cyan sticks. (**B**) Superimposition of the PfNCR1-I and PfNCR1-II structures. The secondary structural elements of PfNCR1-I are colored yellow, whereas those of PfNCR1-II are colored green. The cross-sectional area of the narrowest region of the tunnel is also included. This cross section indicates that residues 1105–1108, which form a short α17 in PfNCR1-I (yellow), block and disconnect the tunnel of this transporter. In the PfNCR1-II structure (green), these residues switch its conformation to form a random coil and also shift away from the central core by approximately 7 Å to open the tunnel. The pink oval indicates the cross section of the narrowest region of the tunnel formed by PfNCR1-II.

The overall conformation of this PfNCR1-II structure (Fig. 2A) is similar to that of PfNCR1-I. Superimposition of these two protomers results in an RMSD of 1.3 Å, indicating that these two PfNCR1 structures represent very different transient conformational states (Fig. 2B). A detailed inspection reveals that the elongated tunnel spanning the outer leaflet of the PPM domain and the PV domain is open (Fig. 2A and fig. S4). Therefore, the conformation of PfNCR1-II most likely represents the open-tunnel form of this transporter. It appears the flexible linkers connecting the C-terminal end of TM7 and N-terminal end of α18 of PV1, and the C-terminal end of TM1 and N-terminal end of α2 of PV1 responsible for the opening and closing of this tunnel. In particular, residues 1105–1108 form a short α17 in PfNCR1-I and block this PfNCR1-I tunnel. In the PfNCR1-II structure, these residues switch its secondary structural conformation to form a random loop. In addition, the entire flexible linker formed by residues 1101–1110 of PfNCR1-II shifts its location and moves away from the central core of the tunnel by approximately 7 Å to open this PfNCR1-II tunnel (Fig. 2B).

Like PfNCR1-I, an extra density is found nearby the ceiling of the PV domain, corresponding to a bound Chl molecule sandwiched between subdomains PV1 and PV2 (Fig. 2A, inset). The location of bound Chl is more or less identical to that of PfNCR1-I. Within 4.5 Å of bound Chl, identical residues, including R90, F387, M398, F1132, A1208, F1213, V1243, L1246, F1247, and H1264, surround this sterol molecule to secure the binding.

### Structure of the PfNCR1-MMV009108 transporter-inhibitor complex

**Fig. 3.**
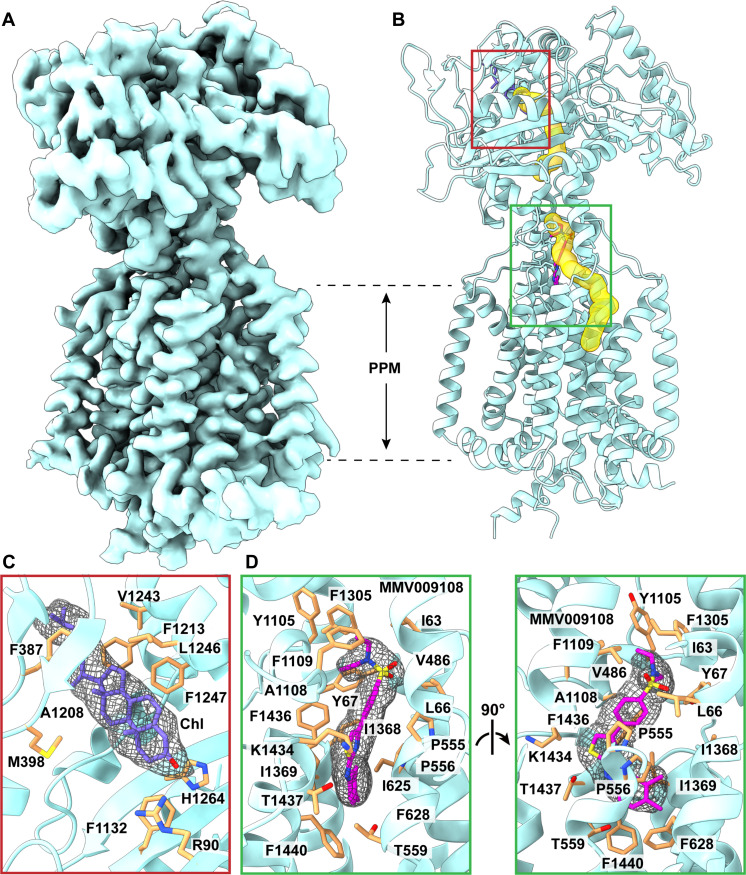
Structure of PfNCR1-MMV009108. (**A**) Cryo-EM map of PfNCR1-MMV009108 at a resolution of 2.98 Å. (**B**) Side view of the ribbon diagram of PfNCR1-MMV009108 viewed in the membrane plane. The structure of PfNCR1-MMV009108 indicates that this transporter forms a channel (yellow) spanning the outer leaflet of the PPM domain and up to the PV domain. Similar to PfNCR1-I, this channel is broken into two halves. (**C**) Chl binding site. This Chl binding site is located between subdomains PV1 and PV2, identical to those found in the PfNCR1-I and PfNCR1-II structures. The bound Chl molecule is in slate sticks, and its cryo-EM density is in gray meshes. Residues R90, F387, M398, F1132, A1208, F1213, V1243, L1246, F1247, and H1264, which are important for Chl binding, are in orange sticks. (**D**) MMV009108 inhibitor binding site. The bound MMV009108 compound is in magenta sticks, and its cryo-EM density is in gray meshes. Residues I63, L66, Y67, V486, P555, P556, T559, I625, F628, Y1105, A1108, F1109, F1305, I1368, I1369, K1434, F1436, T1437, and F1440, which are critical for interacting with MMV009108, are in orange sticks.

PfNCR1 has been shown to be a druggable target (*10*). It is required for maintaining the proper membrane lipid composition necessary for the development of parasites in the blood stage (*10*). A selection study using isolated parasites with resistance-conferring mutations in PfNCR1 has allowed for the identification of three diverse antimalarial MMV small-molecule compounds that directly interact with the PfNCR1 membrane protein. One such small molecule is MMV009108, which leads to the hypersensitivity of *P. falciparum* to saponins (*10*). To elucidate the structural basis of how PfNCR1 and MMV009108 interacts as well as the inhibition mechanism of this MMV compound, we incubated purified PfNCR1 with MMV009108 to form the PfNCR1-MMV009108 complex and then solved the cryo-EM structure of this complex to a resolution of 2.98 Å (Fig. 3, A and B; fig. S8; and table S1). We only observed one class of single-particle images, which represents the MMV009108-bound structure of the PfNCR1 transporter.

The overall structure of PfNCR1-MMV009108 is very similar to that of PfNCR1-I as described above. Superimposition of these two structures gives rise to an RMSD of 0.5 Å (for 871 Cα atoms), suggesting that the secondary structure of the transporter does not change significantly after inhibitor binding. The structure of PfNCR1-MMV009108 depicts a closed tunnel conformation of the transporter, similar to that found in PfNCR1-I (fig. S4). An extra density resembling Chl molecule is observed at the Chl binding site (Fig. 3C). The mode of binding for this Chl is the same as that found in the PfNCR1 structure without the addition of inhibitor. Again, residues R90, F387, M398, F1132, A1208, F1213, V1243, L1246, F1247, and H1264 are responsible for the binding (Fig. 3C).

Our high-quality cryo-EM map also allows us to unambiguously depict the location of bound MMV009108. This inhibitor binds within the tunnel created by the PfNCR1 transporter. Within 4.5 Å from bound MMV009108, residues I63, L66, Y67, V486, P555, P556, T559, I625, F628, Y1105, A1108, F1109, F1305, I1368, I1369, K1434, F1436, T1437, and F1440 are engaged in anchoring this inhibitor molecule (Fig. 3D). On the basis of this structural information, the inhibition mechanism of MMV009108 appears to be due to a physical blockage of the tunnel formed by PfNCR1, disallowing this transporter to export Chl from the PPM of the parasite. It is worth noting that residue A1108 is 3.6 Å away from the six-carbon aromatic ring of MMV009108, contacting this inhibitor via hydrophobic interaction. It has been documented that a mutation on this alanine to a threonine causes the MMV009108 drug to be incapable of inhibiting *P. falciparum*, reversing parasite susceptibility to saponins in the presence of this drug (*10*). Our structure of PfNCR1-MMV009108 is consistent with this inhibitor not binding the transporter in the A1108T mutant, possibly due to steric hinderance (fig. S9).

### Docking calculations

#### Docking of Chl into PfNCR1

The cryo-EM structure of PfNCR1 depicts that this transporter creates two large cavities located at the vacuolar cleft of the PV and outer leaflet of the PPM, respectively. A bound Chl molecule was identified at the large cavity between subdomains PV1 and PV2. The second large cavity is surrounded by TM2, TM3, and TM4, mimicking the SSD binding site at the outer leaflet of the PPM. We suspected that this SSD cavity is able to accommodate Chl. We, therefore, used the AutoDock Vina program (*20*) to elucidate if the SSD cavity located at the PPM is capable of binding Chl. We first docked Chl into the Chl binding site formed between subdomains PV1 and PV2. We observed that Chl was bound at the same location and orientation as identified in the cryo-EM structure of PfNCR1 (fig. S10). We then studied the interaction of PfNCR1 with Chl at the SSD cavity using the same approach. We found that PfNCR1 specifically contacts and houses Chl in this SSD cavity surrounded by TM2 to TM4, indicating that this cavity forms a Chl binding site (fig. S10). The predicted binding affinities of Chl for these two Chl binding sites are −8.7 and −8.5 kcal/mol, respectively. Additionally, the binding affinities for NBD-Chl at these two binding sites (fig. S10) were calculated to be −9.8 and −9.5 kcal/mol, respectively.

#### Docking of inhibitors into PfNCR1

We used the same approach to elucidate PfNCR1-inhibitor interactions based on AutoDock Vina (*20*). We first docked MMV009108 into its binding site that was determined from cryo-EM. We found that PfNCR1 specifically interacts with this inhibitor at the MMV009108 binding site as observed from the PfNCR1-MMV009108 structure (fig. S11). We then studied the interactions of PfNCR1 with the other two inhibitors MMV028038 and MMV019662 using Vina (*20*). Vina indicated that both MMV028038 and MMV019662 specifically interact with PfNCR1 at their corresponding predicted binding sites along the Chl tunnel formed by PfNCR1 (fig. S11). The predicted binding affinities for MMV009108, MMV028038, and MMV019662 with PfNCR1 were calculated to be −13.2, −10.1, and −12.2 kcal/mol, respectively.

### Computational simulations of the PfNCR1 transporter

The gene *pfncr1* (PF3D7_0107500), encoding the PfNCR1 membrane protein, is annotated as a lipid/sterol transporter. On the basis of the structural information, PfNCR1 creates two Chl binding sites and an elongated tunnel spanning these two sites. It is likely that this transporter is capable of shuttling Chl from one binding sites to the other. To elucidate the mechanism of Chl transport, we performed MD (figs. S12 and S13) and TMD (fig. S14) simulations on the PfNCR1 transporter in an explicit lipid bilayer and water environment using Amber (*21*, *22*) and NAMD (*23*).

#### Dynamics of the PfNCR1 transporter

Principal components analysis (PCA) indicates that the first and second eigenvectors, which depict the two most important motions extracted from the MD simulation trajectory, correspond to a rigid-body movement of subdomain PV1 in relation to subdomain PV2 of the PfNCR1 transporter (fig. S13). This rigid-body motion is accompanied by slight movements of the TM helices at the PPM domain. Overall, PCA suggests that the major conformational change of the TM region is the upward shift of the N-terminal half of the TM region (TM1 to TM6). This movement is accompanied by a downward shift of the PV1 subdomain. The rigid-body movement of the PV1 subdomain can be interpreted as the motion to open and close the cleft between subdomains PV1 and PV2. This motion could help promote the transport of substrates via the PfNCR1 transporter.

#### Chl transport pathway

TMD simulations allowed us to observe that the Chl molecule is able to follow the path of the tunnel identified from the cryo-EM structure of PfNCR1 and shuttle from the PPM to the PV region (fig. S14). According to the simulations, Chl first enters the PfNCR1 transporter via the Chl binding site at the SSD cavity surrounded by TM2 to TM4. It then passes through the elongated tunnel connecting the PPM and PV regions, and then reaches PV cleft to the Chl binding site located between subdomains PV1 and PV2. This process is accompanied by an upward shift of TM1 to TM4 and a downward movement of PV1, including α14, β10, and α18. In addition, the loops connecting the TM and PV regions, particularly residues 478–493 (including α15) and residues 1099–1113 (including α17), seem to adjust their locations to accommodate the transport of Chl. TMD simulations indicate that PfNCR1 is capable of exporting Chl from the outer leaflet of the PPM to the PV region.

#### Putative proton transfer pathway

We performed 1-μs MD simulations on PfNCR1-I. We identified two water-accessible pockets, as illustrated in fig. S15. The first pocket, situated at the outer leaflet side of the PPM, is composed of residues D1352, S1370, S1377, S1381, T1443, and S1451. The second pocket, located at the inner leaflet side of the PPM, is formed by residues Y510, D570, D571, D1383, H1384, and H1387. These water-accessible residues likely play a crucial role in forming the proton-relay network for energy coupling. Figure S15 illustrates the detailed putative proton transfer pathway. On the basis of the simulation, protons enter the outer leaflet side of the water-accessible pocket and subsequently transfer through the proton-relay residues D1352, S1370, S1377, S1381, T1443, S1451, D1383, H1384, S1451, and S1381. The pathway also involves passing through residues D1383, H1384, and H1387, enabling entry into the inner leaflet side of the water-accessible pocket, and then to residues D570, D571, and Y510, where these protons finally reach the cytoplasmic region of the parasite.

### Chl efflux assays

**Fig. 4.**
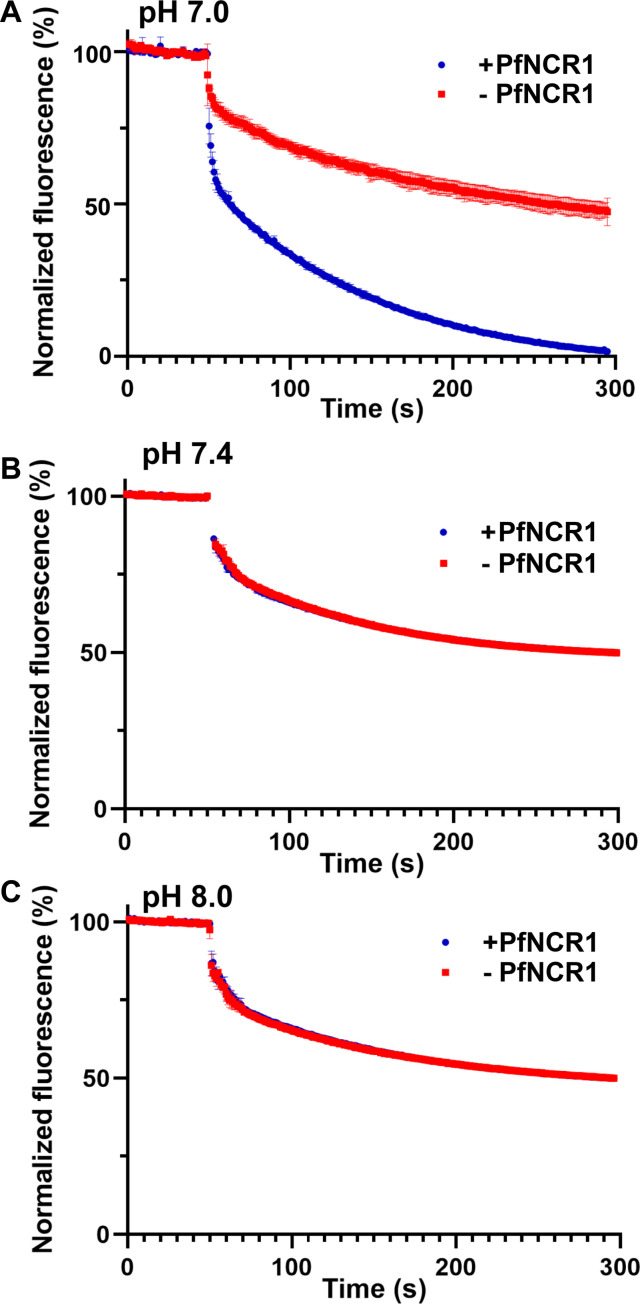
PfNCR1 facilitates the efflux of Chl from mammalian cells. (**A**) At the extracellular pH of 7.0, the efflux of NBD-Chl from the plasma membrane of HEK293 cells expressing PfNCR1 (+ tet) is markedly increased as indicated by the rapid decrease in fluorescence signal when compared with that of the control HEK293 cells (− tet). (**B**) At the extracellular pH of 7.4, the efflux of NBD-Chl from the plasma membrane of HEK293 cells expressing PfNCR1 (+ tet) was found to be no different from that of the control HEK293 cells (− tet), where the fluorescence curves of HEK293 cells with and without expressed PfNCR1 overlap with each other. (**C**) At the extracellular pH of 8.0, the efflux of NBD-Chl from the plasma membrane of HEK293 cells expressing PfNCR1 (+ tet) was found to be no different from that of the control HEK293 cells (− tet), similar to that found in (B). In (A) to (C), all experiments were repeated by three times (blue, HEK293 cells expressing PfNCR1; red, control HEK293 cells without expressing PfNCR1). The small error bars depict SD for *n* = 3. In (A), all fluorescence decay data points from HEK293 cells expressing PfNCR1 at the time interval between 5 and 30 min are significantly different from that of control cells without expressing PfNCR1 (*P* < 0.0001, Student’s *t* test).

HEK293 cells were used to generate an inducible PfNCR1 stable cell line in the presence of tetracycline (+ tet). Western analysis and confocal microscopy indicated that PfNCR1 was expressed on the cell membrane with tet (+ tet) after starvation (fig. S16). This stable line was exposed to fluorescently labeled Chls [NBD-Chl, 22-(*N*-(7-nitrobenz-2-oxa-1,3-diazol-4-yl)amino)-23,24-bisnor-5-cholen-3β-ol] to load the NBD-Chl molecules onto the cell membrane. We then individually conducted comparative analyses of NBD-Chl efflux in the control (− tet) and PfNCR1-expressing HEK293 cells (+ tet) at three different extracellular pHs (pH 7.0, pH 7.4, and pH 8.0). At the extracellular pH of 7.0, a pH lower than that of the intracellular pH, pH 7.4, the efflux of NBD-Chl from the plasma membrane was markedly increased in cells expressing PfNCR1 (+ tet) when compared with that of the control HEK293 cells (− tet) as indicated by the rapid decrease in fluorescence signal (Fig. 4A). The results also indicated that cells expressing PfNCR1 generated an exponential fluorescence decay curve with the rate of 0.39 ± 0.04 s^−1^. This fluorescence decay rate is much faster than that of the control cells (0.10 ± 0.02 s^−1^), suggesting that the expression of PfNCR1 facilitates the export of NBD-Chl from the membrane. However, when the extracellular pH was adjusted to either 7.4 or 8.0, no significant difference in the efflux of NBD-Chl from the plasma membrane was found, as the fluorescence curves of HEK293 cells with and without expressed PfNCR1 overlap with each other (Fig. 4, B and C). For example, at extracellular pH of 7.4, the decay rates of the exponential fluorescence curves for cells expressing PfNCR1 and control cells are 0.12 ± 0.01 s^−1^ and 0.11 ± 0.01 s^−1^, respectively. At extracellular pH of 8.0, these decay rates are 0.10 ± 0.01 s^−1^ and 0.10 ± 0.01 s^−1^. These experimental data suggest that the expression of PfNCR1 facilitates the efflux of Chls from the cell membrane, and that this process is pH dependent where the influx of protons may be a prerequisite to provide the proton motive force (PMF) needed for Chl removal.

## DISCUSSION

Here, we successfully solved high-resolution cryo-EM structures of the PfNCR1 transporter both in the absence and presence of the inhibitor MMV009108. This transporter has been proven to be an attractive antimalarial target, as it resides at the parasite’s PPM and plays an important functional role during intraerythrocytic growth. Knockdown of the *pfncr1* gene or drug inhibition of the PfNCR1 membrane protein is lethal to the parasites and increases susceptibility of the PPM to Chl-intercalating saponin glycosides. PfNCR1 in our structure is seen to bind Chl, which helps explain the observed phenotypic effects. The saponin sensitivity likely reflects aberrant Chl accumulation in the PPM when PfNCR1 is impaired. Digestive vacuoles that form from the parasite membranes are normally devoid of Chl (*10*, *24*) but, in the knockdown/inhibited parasites, form aberrant structures with abnormal membrane curvature, again suggesting Chl accumulation. It seems likely that the role of PfNCR1 is to remove Chl from the PPM. As a consequence, digestive vacuoles that form from the parasite membranes and are normally devoid of Chl (*10*, *24*) form aberrant structures with abnormal membrane curvature. Since previous studies have shown that PfNCR1 resides at regions of contact between the PPM and parasitophorous vacuolar membrane (PVM) (*13*), it seems likely that the role of PfNCR1 is to remove Chl from the PPM. We suggest that PfNCR1 brings the Chl directly to the PVM, taking advantage of the physical proximity of the two membranes. Our cryo-EM structure of PfNCR1 indicates that a bound Chl molecule is found within the cavity created between subdomains PV1 and PV2. Moreover, an elongated tunnel within the transporter directly connects this Chl binding site to the SSD cavity. AutoDock Vina suggests that the SSD cavity is capable of binding Chl, creating a putative Chl binding site. Chl efflux assays indicate that recombinant PfNCR1 expressed in HEK293 cells is able to facilitate the export and removal of Chl from the cell membrane. This observation is further strengthened by TMD simulations, where PfNCR1 is capable of transporting Chl from the PPM to PV domains of PfNCR1 using the tunnel formed within this transporter.

**Fig. 5.**
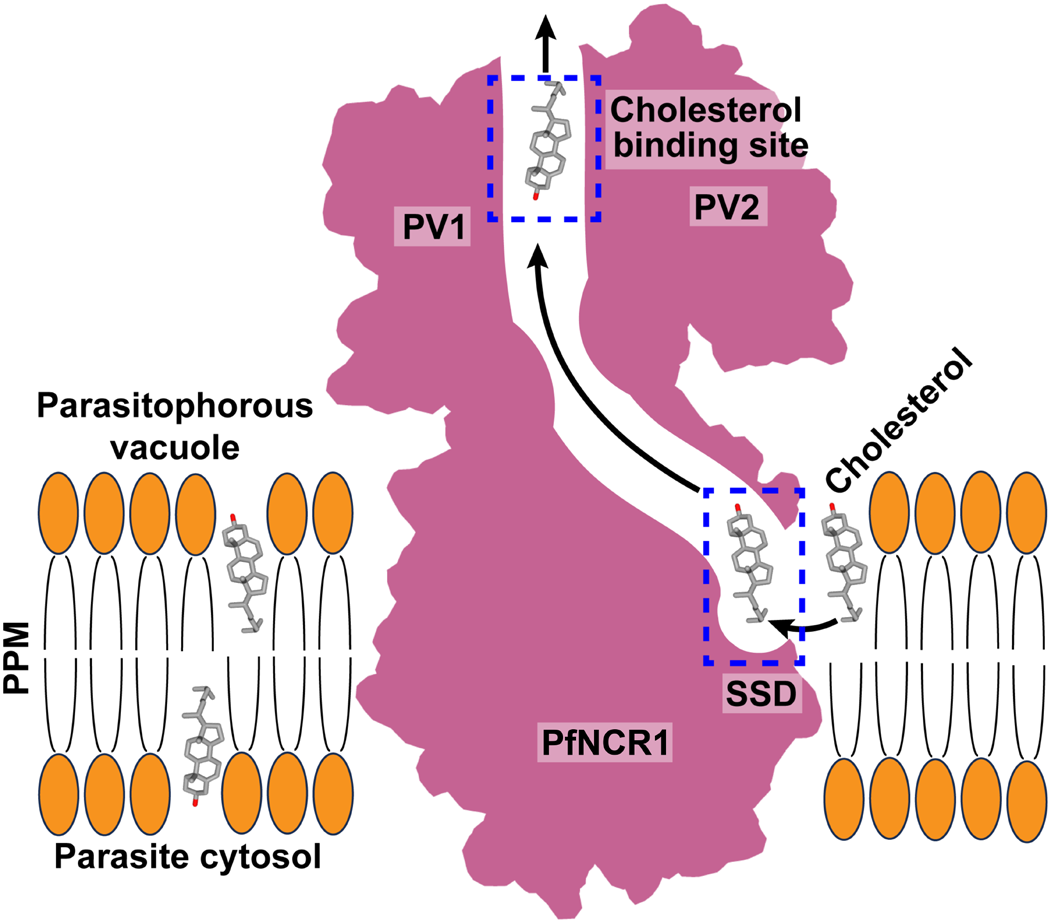
Proposed mechanism for Chl translocation via PfNCR1. This schematic diagram indicates that the PfNCR1 transporter is capable of picking up a Chl molecule from the parasite’s plasma membrane. This Chl molecule will arrive the SSD of PfNCR1 and pass through the tunnel formed by this transporter. The Chl moiety will then reach the Chl binding site located between PV1 and PV2 of the PV domain of PfNCR1 in the PV and be exported out of the transporter.

On the basis of the structural, computational, and biochemical results, we propose that the PfNCR1 transporter captures Chl from the outer leaflet of the PPM, shuttles it to the SSD and PV Chl binding sites, and eventually exports the sterol molecule to the parasitophorous vacuolar space via the Chl tunnel constituted by the transporter (Fig. 5). A cleft surrounded by subdomains PV1 and PV2 of the PV domain forms the Chl binding site. This binding site located at the PV domain probably also marks the exit site of the tunnel, which spans the SSD cavity situated in the outer leaflet of the PPM and up to the parasitophorous vacuolar space. On the basis of the structural information, an additional chaperon and/or adaptor protein that specifically interacts with PfNCR1 may be required to complete Chl transport. Further structural and functional studies are needed to fully define this process.

In the parasite, the PPM and PVM form regions of close contact, which would allow for lipid transfer (*13*). PfNCR1 localizes to these sites, while proteins involved in aqueous solute transport localize to separate domains in the PVM. As a member of the RND superfamily of transporters (*14*), PfNCR1 is likely a PMF-dependent transporter that functions via a substrate/proton antiport mechanism. Therefore, PfNCR1 may get its energy to work against the concentration gradient and remove Chl from the Chl-poor PPM to the Chl-rich PVM using the PMF. This would require the protons being relayed in the opposite direction of Chl efflux. Two independent studies comparing parasite and infected erythrocyte cytoplasmic pH have been done, with similar results (*25*, *26*). The pH external to the parasite was estimated at 7.1 (one measured 6.9 close to the parasite), and the pH inside the parasite was 7.3. Thus, a gradient of 0.2 to 0.4 pH units exists across the parasite surface so the directionality of proton transfer should be the influx of protons from the erythrocyte and contiguous parasitophorous vacuolar space to the cytoplasm of the parasite. Our MD simulations data suggest a putative proton-relay network that facilitates the transport of protons in this direction. Under these pH conditions, our Chl efflux assays indicate that PfNCR1 is able to mediate the efflux of Chl from the parasite’s plasma membrane to the parasitophorous vacuolar space. This would be similar to what is seen in the bacterial RND superfamily of transport systems, including the hopanoid lipid transporter *Burkholderia multivorans* HpnN (*27*) and the trehalose monomycolate lipid transporter *Mycobacterium smegmatis* MmpL3 (*28*, *29*), where these transporters are responsible for bacterial cell envelope and cell membrane biogenesis. It is possible that PfNCR1 is not strictly a PMF-driven transporter. Inhibition of PfATP4, a sodium pump, yields a saponin sensitivity phenotype similar to that of inhibition of PfNCR1 (*30*). The sodium gradient could plausibly contribute to PfNCR1 transport, in addition to or instead of the PMF. As precedent, the *Drosophila* family member Patched has been proposed to use an alkali metal cation to drive Chl transport (*31*). A similar observation has been seen with the *Alcanivorax borkumensis* YdaH transporter, a member of the PMF-dependent AbgT family of transporters (*32*), where YdaH was found to be both PMF and Na^+^ dependent (*33*).

PfNCR1 is an attractive antimalarial drug target. Its druggability has been demonstrated by the inhibitory efficacy of three MMV drug candidates. When these compounds directly interact with PfNCR1, they elevate the sensitivity of *P. falciparum* to saponin lysis. Our data demonstrate that these drugs specifically bind to functional motifs within the transporter and that their inhibition mechanism is via a direct blockage of the PfNCR1 tunnel, hindering the function of this transporter to traffic Chl. As the expression of PfNCR1 is capable of mediating the parasite’s resistance to saponin, it raises the question whether PfNCR1 can also function as a drug efflux pump specific to steroidal antimalarials, although it is likely that the removal of Chl from the membrane is what makes the membrane insensitive to saponin. A similar phenomenon has been seen in the AbgT family of bacterial membrane proteins, where these transporters have been found to be capable of exporting the folate synthesis catabolite *p*-aminobenzoyl-glutamate (PABA) from bacterial cells (*33*–*35*). However, their canonical function is to perform as antibiotic efflux pumps and extrude sulfonamides to mediate bacterial resistance to these antimetabolite drugs (*33*–*35*).

Our study is a significant advancement in the field of antimalarial therapeutics as it defines vulnerable functional properties in the structure of PfNCR1 in the context of its herein identified substrate Chl. These findings can be used to develop antimalarial drugs that are unlikely to be sensitive to current parasite drug resistance mechanisms.

## MATERIALS AND METHODS

### Expression and purification of PfNCR1

The codon-optimized DNA of full-length PfNCR1 from *P. falciparum* PF3D7_0107500 was synthesized and cloned into pcDNA3.1-N-DYK (GenScript) in frame with a thrombin cleavage site and Twin-Strep-tag at the C terminus. The resulting plasmid was confirmed by the Sanger method of DNA sequencing.

The human embryonic kidney Expi293F (Thermo Fisher Scientific) cells were cultured in Expi 293 Expression Medium (Gibco) at 37°C supplemented with 8% CO_2_. The PfNCR1 protein was expressed using a transient expression system with the following procedures. The purified pcDNA3.1-N-DYK plasmid expressing full-length PfNCR1 was mixed with cationic liposomes (Transfection Reagent I, Avanti Polar lipid) at a 1:10 (DNA:liposome) (w/w) ratio in Opti-MEM I reduced serum medium (Gibco) and incubated at room temperature for 15 min. The resulting lipoplexes were added to cells (cell density of 2.5 × 10^6^ to 3 × 10^6^ cells/ml) at a final DNA concentration of 1 mg/liter. To boost protein expression, valproic acid (Sigma) was added at a final concentration of 3 mM after 18 to 24 hours and allowed to proceed for total 72 hours.

Cells were collected and resuspended in lysis buffer [20 mM Hepes-NaOH (pH 7.5) and 150 mM NaCl] and disrupted with a French pressure cell. The membrane fraction was collected and washed once with the lysis buffer. The membrane protein was then solubilized in 1% (w/v) glycol-diosgenin (GDN) for 3 hours at 4°C. Insoluble material was removed by ultracentrifugation at 100,000*g*. The extracted protein was applied to a Strep-Tactin affinity column (IBA Lifesciences) and washed twice with 15 column volumes of lysis buffer supplemented with 0.01% GDN. The PfNCR1 protein was eluted by adding 4 mM desthiobiotin. The purity of the PfNCR1 protein (>90%) was judged using SDS–polyacrylamide gel electrophoresis stained with Coomassie Brilliant Blue (fig. S17). To enhance sample homogeneity, the PfNCR1 protein was further purified using a Superose 6 column (GE Healthcare) equilibrated with 20 mM tris-HCl (pH 7.5), 100 mM NaCl, and 0.005% GDN. The purified protein was then concentrated to a final concentration of 7 mg/ml.

### Electron microscopy sample preparation

The PfNCR1 protein embedded in GDN detergent micelles was concentrated to 7 mg/ml. A 2.5-μl sample was applied to glow-discharged holey carbon grids (Quantifoil Cu R1.2/1.3, 300 mesh), blotted for 5 s, and then plunge frozen in liquid ethane using a Vitrobot (Thermo Fisher Scientific). The grids were transferred into cartridges. For high-resolution data collection, the sample grids were loaded into a Titan Krios cryo–electron microscope operated at 300 kV equipped with Gatan BioQuantum imaging filter (GIF) and a K3 summit direct electron detector (Gatan). The micrographs were recorded using SerialEM (*36*) with counting mode at nominal ×81,000 magnification corresponding to a calibrated pixel size of 1.07 Å (super-resolution, 0.535 Å/pixel) and a defocus range of −1 to −1.5 μm. To remove inelastically scattered electrons, the slit width was set to 20 eV. Each micrograph was exposed with a total specimen dose between 33 and 35.9 e^−^/Å^2^, and ~38 frames were captured per specimen area.

For the PfNCR1-MMV009108 inhibitor complex, a 40 μM PfNCR1 sample was incubated with 50 μM MMV009108 for 2 hours to form the PfNCR1-MMV009108 complex. A 1-μl sample was applied to glow-discharged holey carbon grids (UltrAufoil Au R1.2/1.3, 300 mesh) and blotted for 5 s, and a 1.7-μl sample was applied to the grid again with 3-s blotting and then plunge-frozen in liquid ethane using a Vitrobot (Thermo Fisher Scientific). The procedures for high-resolution cryo-EM data collection were the same as those described above. Each micrograph was exposed with a total specimen dose of 40.5 e^−^/Å^2^, and 45 frames were captured per specimen area.

### Cryo-EM data processing

The micrographs of PfNCR1 were aligned by using patch-based motion correction for beam-induced motion using CryoSPARC (*37*). The contrast transfer function (CTF) parameters of the micrographs were determined using Patch CTF (*38*). After manual inspection and sorting to discard poor micrographs, ∼2000 particles of PfNCR1 were manually picked to generate templates for automatic picking. Initially, 6,977,953 particles were selected after autopicking in CryoSPARC. Several iterative rounds of 2D classifications followed by ab initio and heterogeneous 3D classifications were performed to remove false picks and classes with unclear features, ice contamination, or carbon. The 3D classification analysis was then used, resulting in two distinct classes. Nonuniform refinement followed by local refinement with nonuniform sampling resulted in 3.11-Å- and 3.81-Å-resolution cryo-EM maps for PfNCR1-I and PfNCR1-II based on gold standard Fourier shell correlation (FSC 0.143).

### Model building and refinement

Model building of PfNCR1-I was based on the 3.11-Å cryo-EM map. The predicted modeling structure of PfNCR1 generated by AlphaFold (*39*) was fitted into the density map using Chimera (*40*). Subsequent model rebuilding was performed using Coot (*41*). Structure refinements were performed using the phenix.real_space_refine program (*42*) from the PHENIX suite (*43*). The final atomic model was evaluated using MolProbity (*44*). Statistics associated with data collection, 3D reconstruction, and model refinement are included in table S1.

The PfNCR1-MMV009108 structural model at 2.98-Å resolution was built based on the PfNCR1-I structure. A 3D conformer of the inhibitor MMV009108 (PubChem CID: 2297659) was processed using phenix.elbow implemented in PHENIX (*43*). Structural refinements were done using the same approach as described above (table S1).

### Molecular docking

The program AutoDock Vina (*20*) was used to predict the binding modes of Chl and the three PfNCR1 inhibitors, including MMV009108, MMV028038, and MMV019662. The PfNCR1-I structure (with the Chl molecule removed) was used for dockings. The protein was set as a rigid structure, whereas the conformation of each antibiotic molecule was optimized via all modeling and docking procedures. For each inhibitor, the results were ranked on the basis of predicted free binding energy, where the one with the highest binding affinity was recorded.

### MD simulations

The protonation states of the titratable residues of the PfNCR1 transporter were determined using the H++ server (http://newbiophysics.cs.vt.edu/H++/). The cryo-EM structure of PfNCR1-I was used as the template for the PfNCR1-Chl structure. We also removed the bound Chl ligand from the PfNCR1-I structure to form the template for apo-PfNCR1. These two structures were individually immersed in an explicit lipid bilayer consisting of 1-palmitoyl-2-oleoyl-sn-glycero-3-phosphocholine (POPC), 1-palmitoyl-2-oleoyl-sn-glycero-3-phosphatidylethanolamine (POPE), 1-palmitoyl-2-oleoyl-sn-glycero-3-phospho-L-serine (POPS), and Chl with a molecular ratio of 25:5:5:1 using the CHARMM-GUI Membrane Builder webserver (http://www.charmm-gui.org/?doc=input/membrane). A water box with dimensions of 104.9 Å × 106.7 Å × 170.0 Å was used. NaCl (150 mM) and extra neutralizing counter ions were added for these simulations. The total number of atoms was 144,403 and 144,432 for the apo-PfNCR1 and PfNCR1-Chl systems, respectively. The Antechamber module of AmberTools was used to generate parameters for Chl using the general AMBER force field (GAFF) (*21*, *22*). The partial charges of Chl were calculated using ab initio quantum chemistry at the HF/6-31G* level (GAUSSIAN 16 program) (Gaussian Inc., Wallingford). The RESP charge-fitting scheme was used to calculate partial charges on the atoms (*45*). The tleap program was used to generate parameter and coordinate files using the ff14SB and Lipid17 force field for both the protein and lipids. The PMEMD.CUDA program implemented in AMBER18 (AMBER 2018, UCSF) was used to conduct MD simulations. The simulations were performed with periodic boundary conditions to produce isothermal-isobaric ensembles. Long-range electrostatics were calculated using the particle mesh Ewald (PME) method (*46*) with a 10-Å cutoff. Before production runs, energy minimization of these systems was carried out. Subsequently, the systems were heated from 0 to 303 K using Langevin dynamics with 1 ps^−1^ collision frequency. During heating, the PfNCR1 transporter was position-restrained using an initial constant force of 500 kcal mol^−1^ Å^−2^ and weakened to 10 kcal mol^−1^ Å^−2^ to allow for the movement of lipid and water molecules. Then, the systems went through 5-ns equilibrium MD simulations. Finally, a total of 1-ms production MD simulations was conducted. During simulations, the coordinates were saved in every 500 ps for analysis. All systems were well equilibrated after 100-ns simulations according to RMSDs of the transporter’s Cα atoms. Trajectories (100 ns to 1 ms) of each system were used for root mean square fluctuation (RMSF) and PCA (*47*, *48*). GROMCAS analysis tools were used for the MD simulation trajectory analysis (*49*).

### TMD simulations

TMD was performed using the NAMD program (*23*) with the same AMBER force field parameters as described above. In the simulations, we selected the heavy atoms of Chl to be guided toward the target position (from the PPM domain to the PV domain) by the application of steering forces. The root mean square (RMS) distance between the current coordinates and the target structure was calculated at each timestep. The force on each selected atom was given by a gradient of potential as a function of the RMS values. The system was gone through energy minimization, heating, and 5-ns equilibrium MD simulations. Then, TMD simulation was performed for 5 ns based on the MD equilibrated coordinates. A value of 500 kcal mol^−1^ Å^−2^ was used as an elastic constant for TMD forces during the simulations.

### Generation of PfNCR1-expressing stable cell line

Wild-type pfNCR1, containing a C-terminal Strep epitope, was subcloned into a pcDNA5/FRT/TO vector (Invitrogen). The stable tetracycline-inducible Strep-pfNCR1 HEK293 Flp-In T-REx expression cell line was generated by cotransfection of the Strep-pfNCR1-pcDNA5 expression construct and the pOG44 plasmid using Lipofectamine 2000 (Invitrogen). Stable transfectants were selected using hygromycin B (Invitrogen) and blasticidin (Gibco). The final single hygromycin-resistant, blasticidin-resistant foci were isolated and expanded to generate individual clonal cell lines. Following the manufacturer’s protocol, the cells were cultured in Dulbecco’s minimum essential medium (DMEM) supplemented with 5% fetal bovine serum (FBS) and 1% penicillin-streptomycin (Invitrogen).

### NBD-Chl export

Tetracycline induction at a concentration of 1000 ng/ml was applied for 40 hours to prompt the expression of PfNCR1. Following overnight starvation (16 hours) in serum-free, tetracycline-free DMEM to deplete endogenous Chl, PfNCR1-expressing HEK293 Flp-In T-REx cells were treated with 2 mM methyl β-cyclodextrin (MCD) and 20 μM NBD-Chl for 2 hours. The expression and localization of PfNCR1 induced by tetracycline (+ tet) were confirmed by Western blot and confocal microscopy analyses after starvation (fig. S16). Next, cells were removed from the plate using Versene (Invitrogen), washed three times with Hepes-Tyrode’s buffer (pH 7.4) (10 mM Hepes, 12 mM NaHCO_3_, 130 mM NaCl, 5 mM d-glucose, 5 mM KCl, 0.4 mM NaHPO_4_, and 1 mM MgCl_2_), and diluted to 1 × 10^6^ cells per milliliter.

A 1 ml of the cell suspension containing 1 × 10^6^ cells was transferred into a quartz cuvette (Starna Cells Inc.). NBD-Chl efflux was recorded as a stirred suspension at 20°C using a fluorometer from Photon Technology International Inc. at 1-s intervals for 300 s total. The first 50 s were set as the background, and the response to pH changes was measured for 250 s. The excitation and emission wavelengths were 469 and 537 nm, respectively. The pHs of the Hepes-Tyrode’s buffer were adjusted accordingly to 7.0, 7.4, and 8.0 (10 μl of 1 M dithionite in H_2_O was used to adjust a 1-ml Hepes-Tyrode’s buffer pH to 7.0; 10 μl of 1 M dithionite in 0.1 M NaOH was used to adjust a 1-ml Hepes-Tyrode’s buffer to 7.4; 10 μl of 1 M dithionite in 0.5 M NaOH was used to adjust a 1-ml Hepes-Tyrode’s buffer pH to 8.0). These pH values were confirmed by a pH meter (Thermo Fisher Scientific) and pH indicator strips (Cytiva). Proper controls, representing the no tetracycline (− tet) induction group, were incorporated into each experiment. Three independent experiments were performed for every condition.

### Immunostaining

Cells were plated on poly-leucine–coated glass coverslips. Both HEK293 Flp-In T-REx cells expressing PfNCR1 (+ tet) and control cells (− tet) were washed three times with Dulbecco’s phosphate-buffered saline (DPBS) and then fixed in 4% paraformaldehyde for 15 min at room temperature. After blocking in 5% donkey serum in DPBS-T (Tween 20 containing DPBS), cells were incubated with mouse anti-strep primary antibodies overnight at 4°C and then anti-mouse Alexa Fluor 594 secondary antibodies for 1 hour at room temperature. The coverslips were mounted using ProLong Gold Antifade Mountant (Invitrogen) after staining cells with 4′,6-diamidino-2-phenylindole (DAPI) at room temperature for 15 min. Fluorescence images were collected on a Leica HyVolution SP8 confocal microscope (Leica Microsystems, Wetzlar, Germany). The laser intensity, filter, and image size (2048 × 2048) were kept the same for all confocal experiments. The zoom factor was kept at 1 for all experiments. The gain value for each channel was maintained at the same value for each experiment.
