# Supplementary material for: The Plasmodium falciparum NCR1 transporter is an antimalarial target that exports cholesterol from the parasite’s plasma membrane
Source: Sci Adv. 2024 Dec 18;10(51):eadq6651. doi: 10.1126/sciadv.adq6651 (PMC11654669; doi:10.1126/sciadv.adq6651)
Supplement: Supplementary file 1 — Figs. S1 to S17 Table S1 [file sciadv.adq6651_sm.pdf]

Science Advances
AAAS

Supplementary Materials for

The *Plasmodium falciparum* NCR1 transporter is an antimalarial target that exports cholesterol from the parasite's plasma membrane

Zhemin Zhang *et al.*

Corresponding author: Edward W. Yu, edward.w.yu@case.edu

Sci. Adv. **10**, eadq6651 (2024)
DOI: 10.1126/sciadv.adq6651

This PDF file includes:

Figs. S1 to S17
Table S1

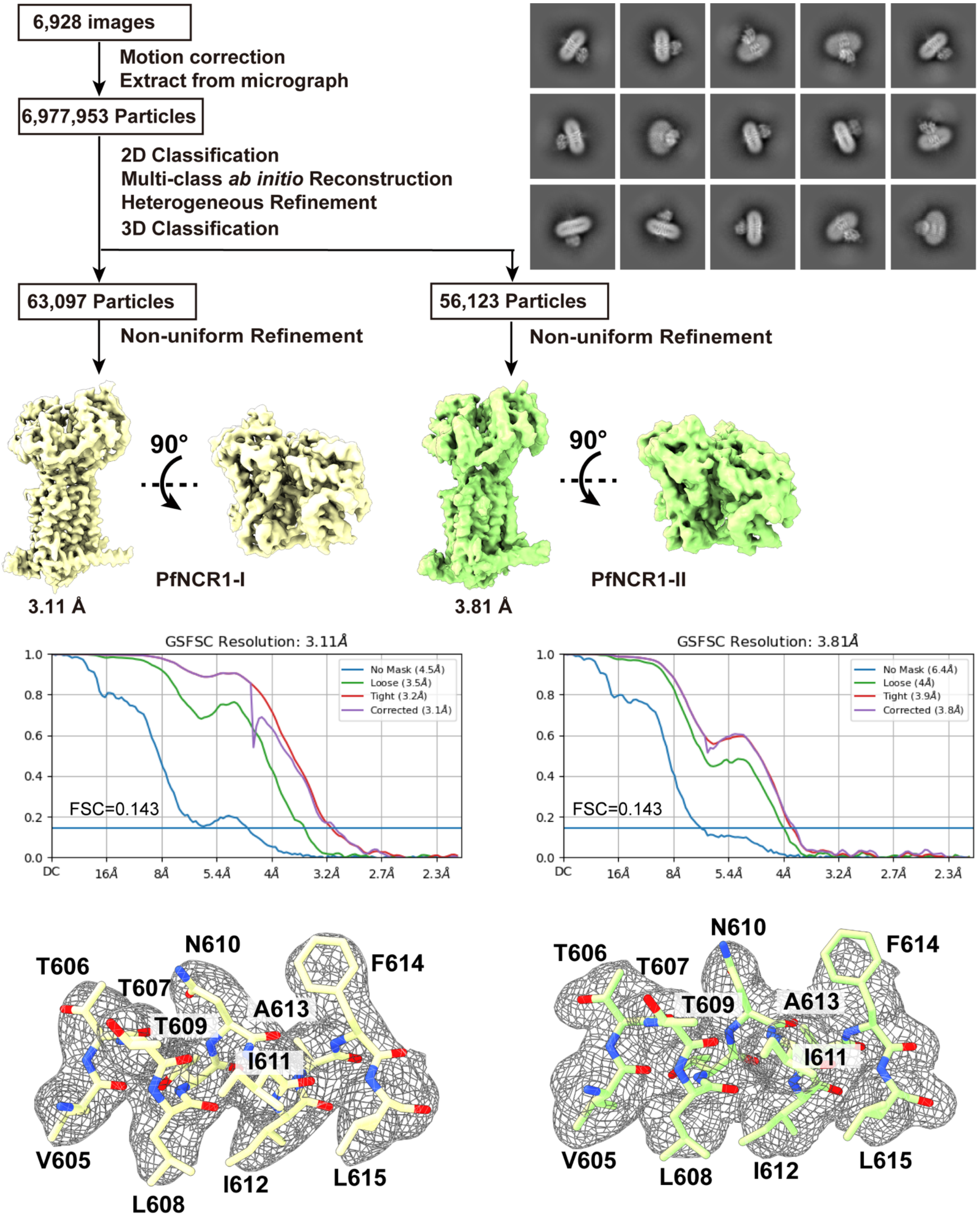

Fig. S1. PfNCR1 data processing workflow. (A) Data processing workflow of PfNCR1-I and PfNCR1-II. Side and top views of the PfNCR1-I and PfNCR1-II density maps. (B) Representative 2D classes of PfNCR1. (C, D) Gold-Standard Fourier shell correlation (GS-FSC) curves of PfNCR1-I and PfNCR1-II. (E, F) Local EM density maps of PfNCR1-I and PfNCR1-II.

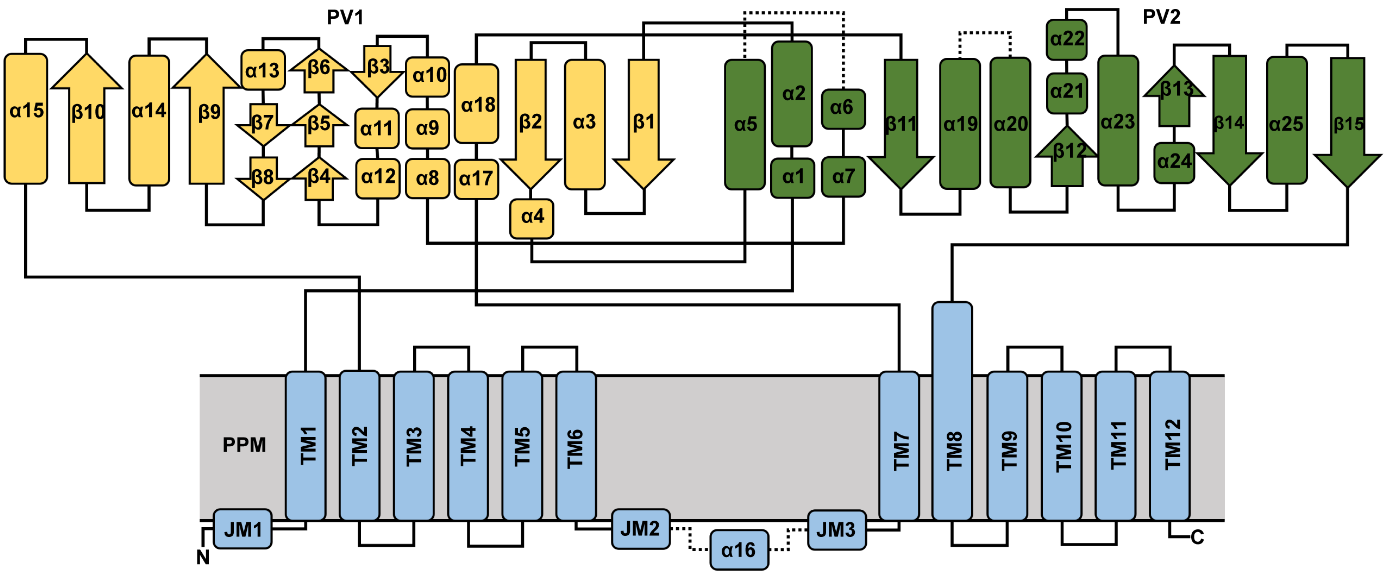

Secondary structure	Residue range	Secondary structure	Residue range
JM1	4-34	TM2	494-514
TM1	36-56	TM3	525-549
α1	63-67	TM4	555-582
α2	73-84	TM5	588-619
β1	89-97	TM6	623-659
α3	104-119	JM2	664-673
β2	121-123	α16	723-735
α4	124-127	JM3	1058-1073
α5	142-161	TM7	1075-1098
α6	295-298	α17	1105-1108
α7	311-313	α18	1114-1125
α8	321-324	β11	1131-1138
α9	334-336	α19	1140-1149
α10	339-344	α20	1185-1200
β3	346-348	β12	1204-1208
α11	349-352	α21	1210-1217
α12	365-368	α22	1221-1223
β4	380-382	α23	1227-1240
β5	388-390	α24	1242-1247
β6	394-398	β13	1251-1254
α13	399-402	β14	1260-126
β7	407-411	α25	1273-1286
β8	414-419	TM8	1301-1334
β9	422-429	TM9	1338-1361
α14	436-452	TM10	1368-1394
β10	472-477	TM11	1400-1431
α15	479-490	TM12	1435-1466

Fig. S2. Secondary structural topology and elements of PfNCR1. The topology was constructed based on the cryo-EM structure of PfNCR1. The transmembrane helices (TMs) are colored cyan. The secondary structural elements of the parasitophorous subdomains PV1 and PV2 are colored yellow and green, respectively. The missing residues are represented by dotted lines. Residues making up these secondary structural elements are listed.

A

Ranking	PDB ID	Z-score	rmsd	# eq res	# res	% id	Protein
1	7k65	13.9	1.8	113	1005	24	mPTCH1 (Mus musculus)
2	6rvd	12.5	1.7	113	978	24	hPTCH1 (Homo sapiens)
3	7dfw	12.4	1.7	114	958	26	hNCR1 (Homo sapiens)
4	5u74	11.8	2.2	110	900	24	hNPC1 (Homo sapiens)
5	7n4u	11.8	1.7	114	953	26	hNCR1 isoform 2 (Homo sapiens)

Z-score: sum of the equivalent C-C distances between two proteins' residues
rmsd: root mean square deviation (Å)
eq res: number of equivalent residues
res: total number of residues
% id: % of identity

B

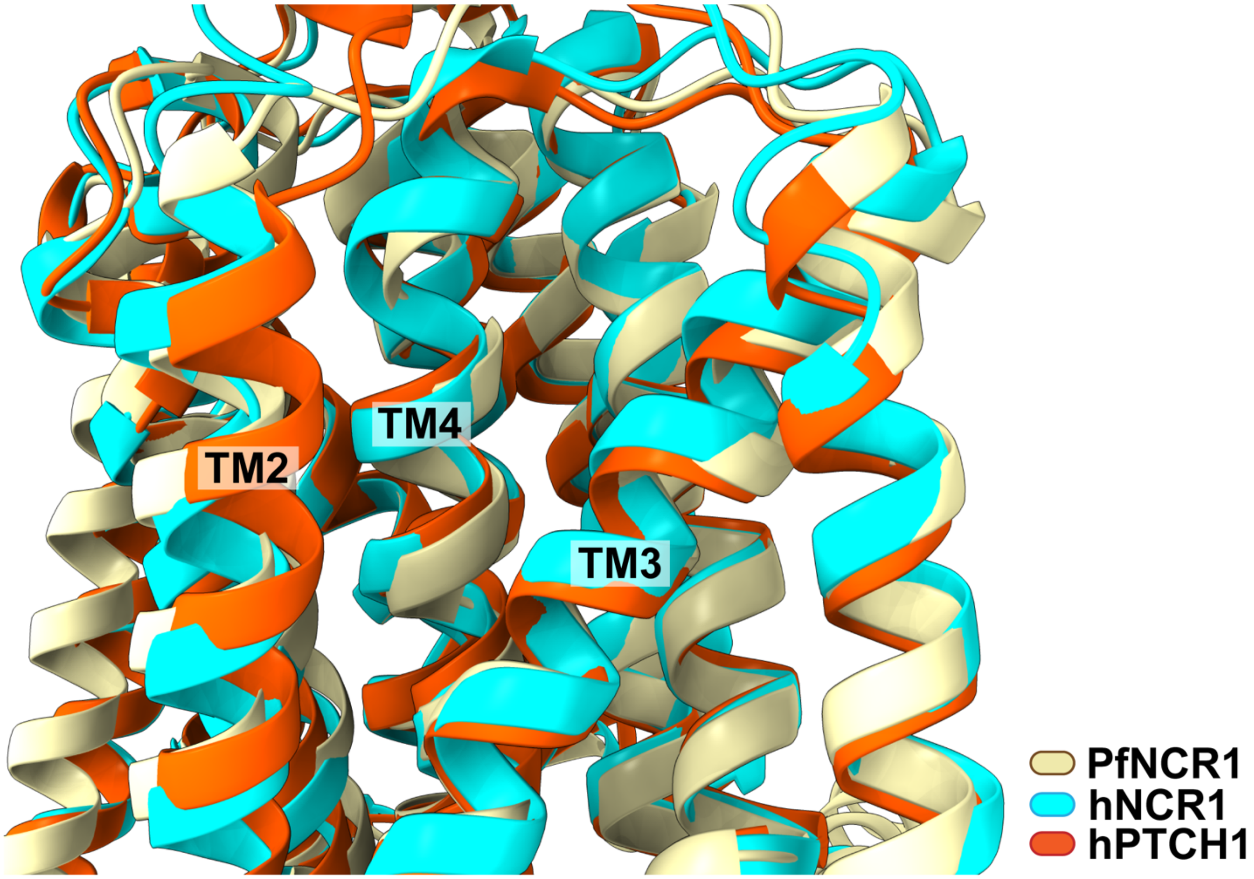

Fig. S3. Structural and sequence alignments of the SSD of PfNCR1 with its closest homolog proteins. (A) The DALI program depicts the top five membrane proteins homologous to PfNCR1. (B) Structural alignments of the SSDs of hPTCH1 and hNCR1 with that of PfNCR1, indicating that TMs 2-4 form the SSD capable of binding sterols and cholesterols.

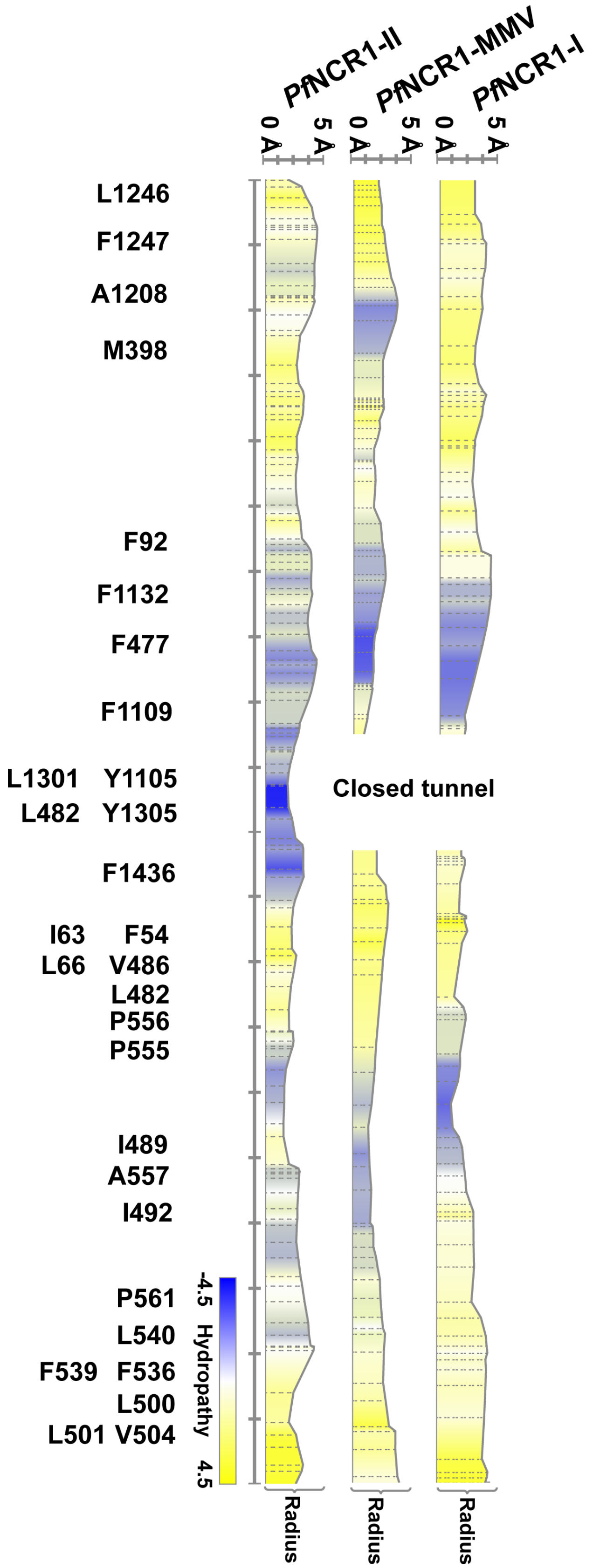

Fig. S4. Tunnel formed by the PfNCR1 transporter. This figure indicates calculated radii and important amino acids lining the tunnels formed by PfNCR1-I, PfNCR1-II and PfNCR1-MMV009108 (PfNCR1-MMV). The figure also indicates that the structure PfNCR1-II forms an open tunnel, whereas both the structures of PfNCR1-I and PfNCR1-MMV009108 depict closed tunnels. Residues L482, L1301, Y1305 and Y1105 are responsible for opening and closing the PfNCR1 tunnel (yellow, less hydrophobic; blue, more hydrophobic).

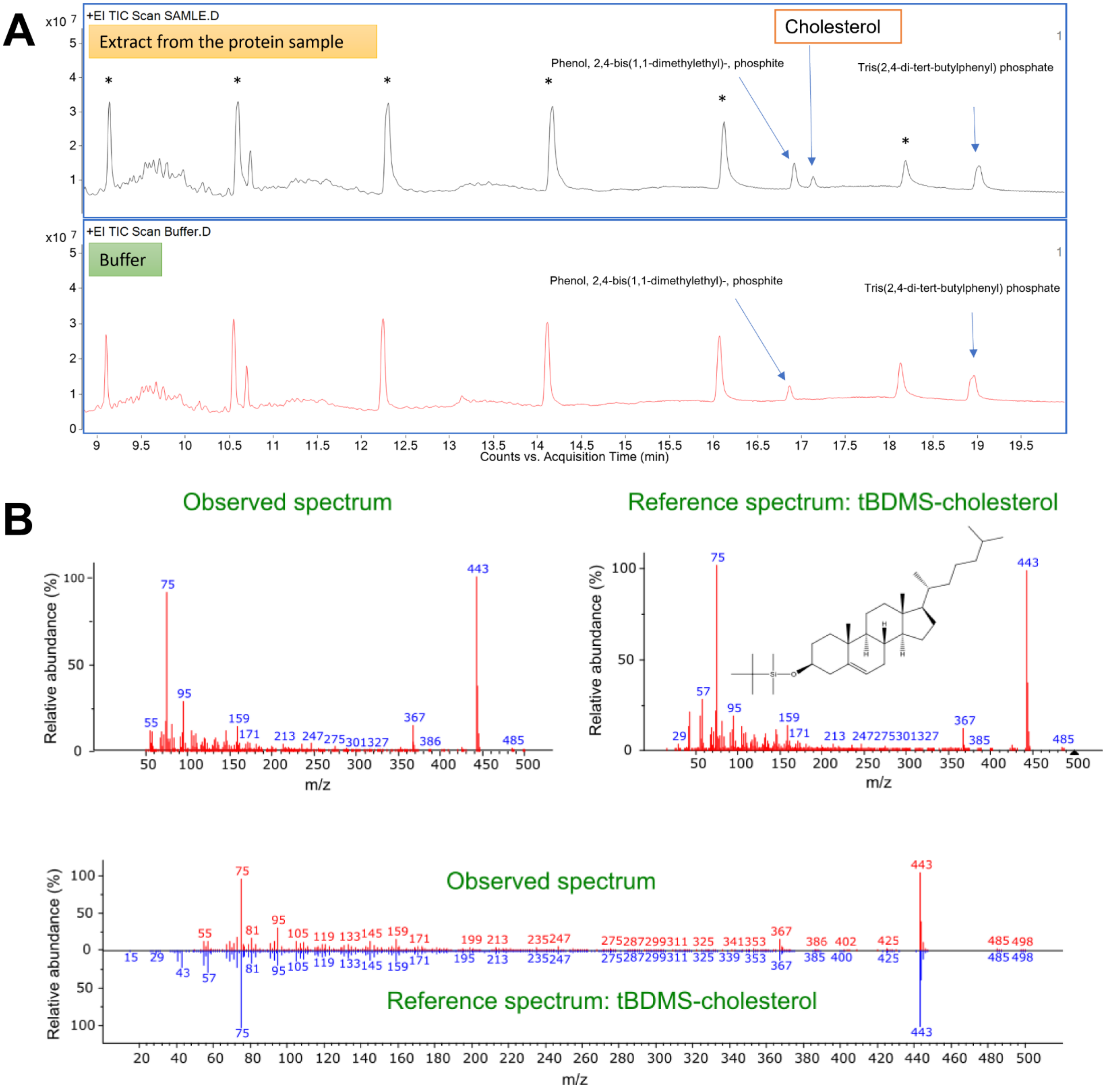

Fig. S5. GC-MS spectra. (A) GC-MS spectra of extract from the purified PfNCR1 protein sample (upper) and buffer of the sample (lower). (B) Comparasion of the observed and reference spectra. These spectra lead to the identification bound chlosterol in the PfNCR1 protein.

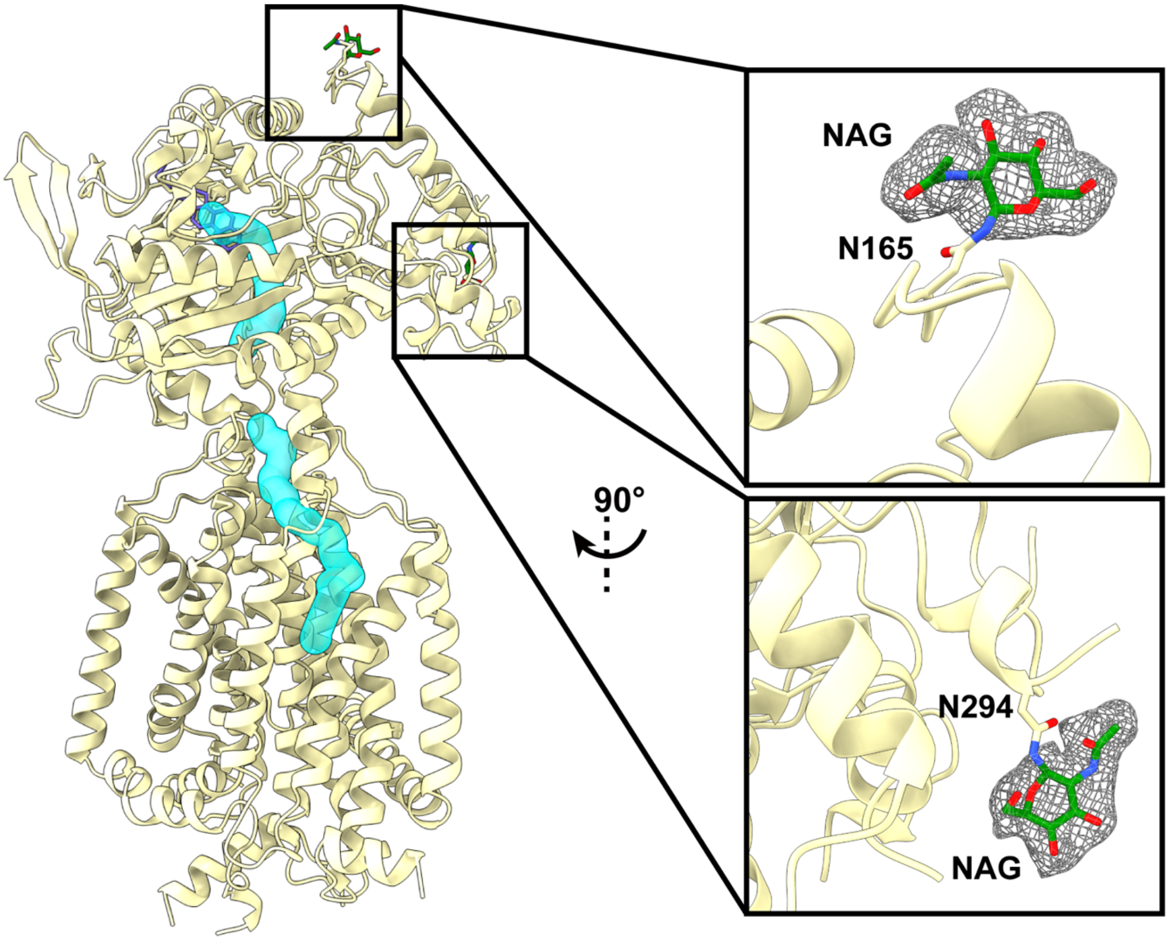

Fig. S6. Cryo-EM densities of the two glycosylation sites of PfNCR1. This figure includes thee secondary structural elements (yellow) of the PfNCR1 transporter. The tunnel formed by PfNCR1 is colored cyan. The cryo-EM densities showing the modifications of N165 and N294 are in grey meshes. Based on the cryo-EM densities, each asparagine is connected to a NAG moiety.

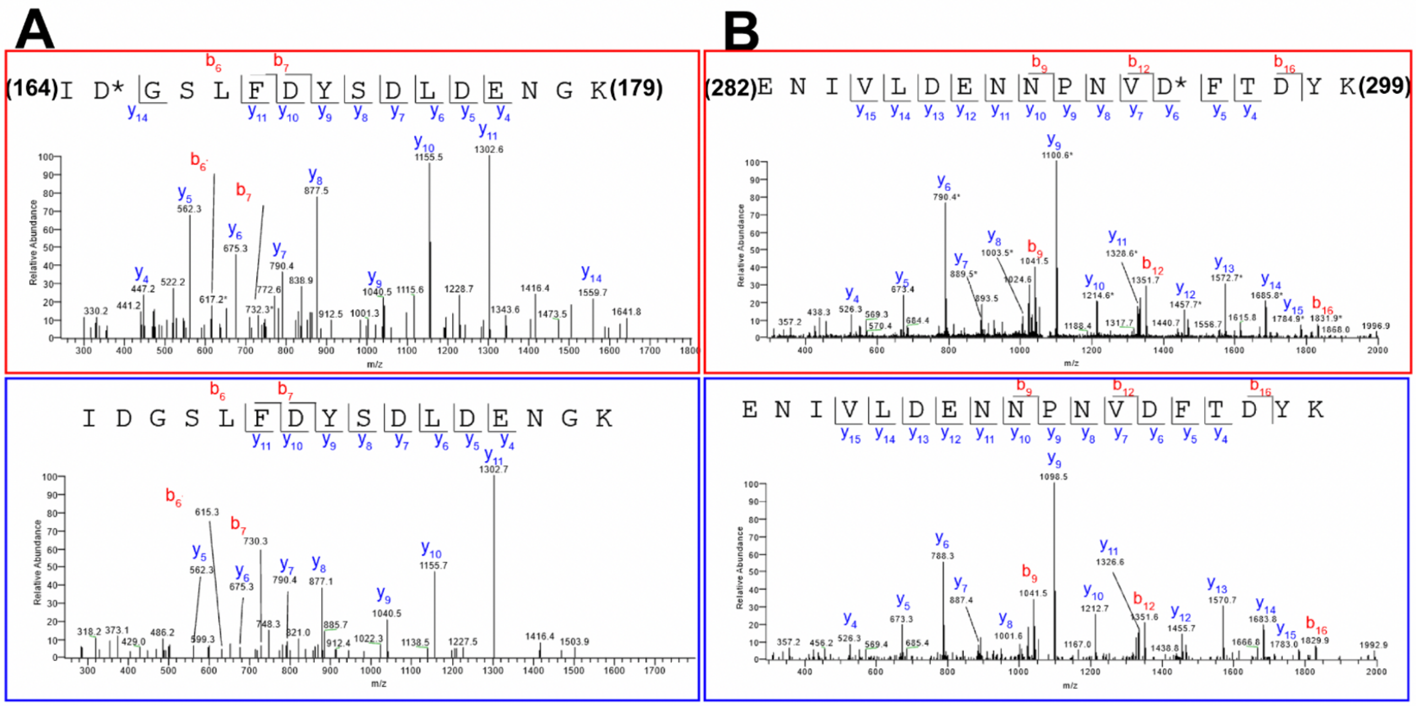

Fig. S7. LC-MS-MS spectra of PfNCR1. (A) Identified glycosylation site at residue N165. (B) Identified glycosylation site at residue N294. In both (A) and (B), the upper panel of the spectra are shown in the presence of $H_2{}^{18}O$, whereas the lower panel of the spectra are shown in the presence of $H_2{}^{16}O$. The amino acid sequence assigned from y-series ions is derived from the C-terminus of the peptide, and the amino acid sequence assigned from b-series ions is derived from the N-terminus of the peptide. The corresponding spectra of the glycopeptide treated with PNGase F are shown. The glycosylated asparagine residue has been converted to aspartic acid by enzymatic reaction. Its signal is shifted by +2 mass units (compared to the corresponding peptide incorporating ^{16}O) as a result of ^{18}O incorporation.

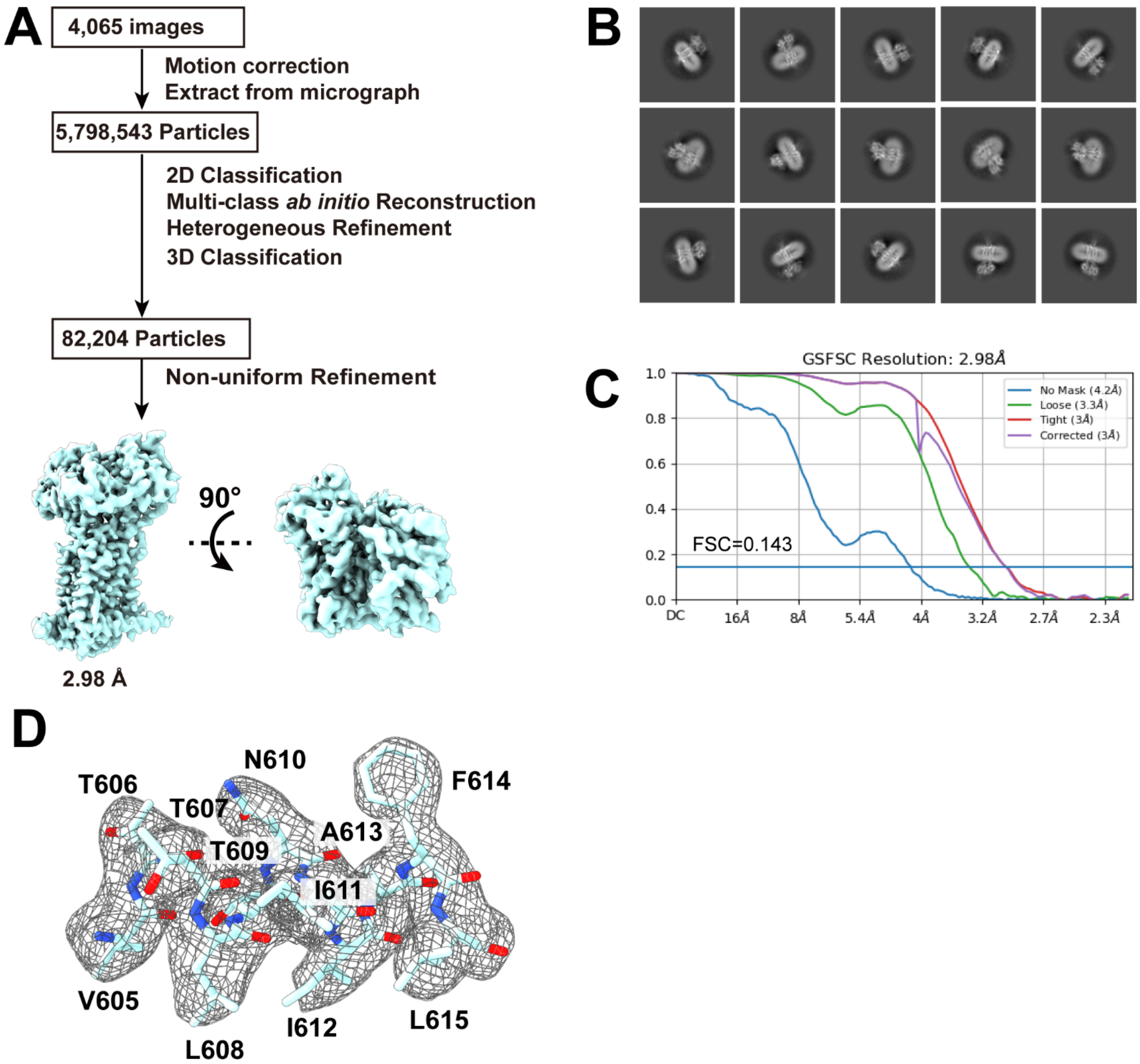

Fig. S8. PfNCR1-MMV009108 data processing workflow. (A) Data processing workflow of PfNCR1-MMV009108. Side and top views of the PfNCR1-MMV009108 density map. (B) Representative 2D classes of PfNCR1. (C) Gold-Standard Fourier shell correlation (GS-FSC) curve of PfNCR1-MMV009108. (D) Local EM density map of PfNCR1-MMV009108.

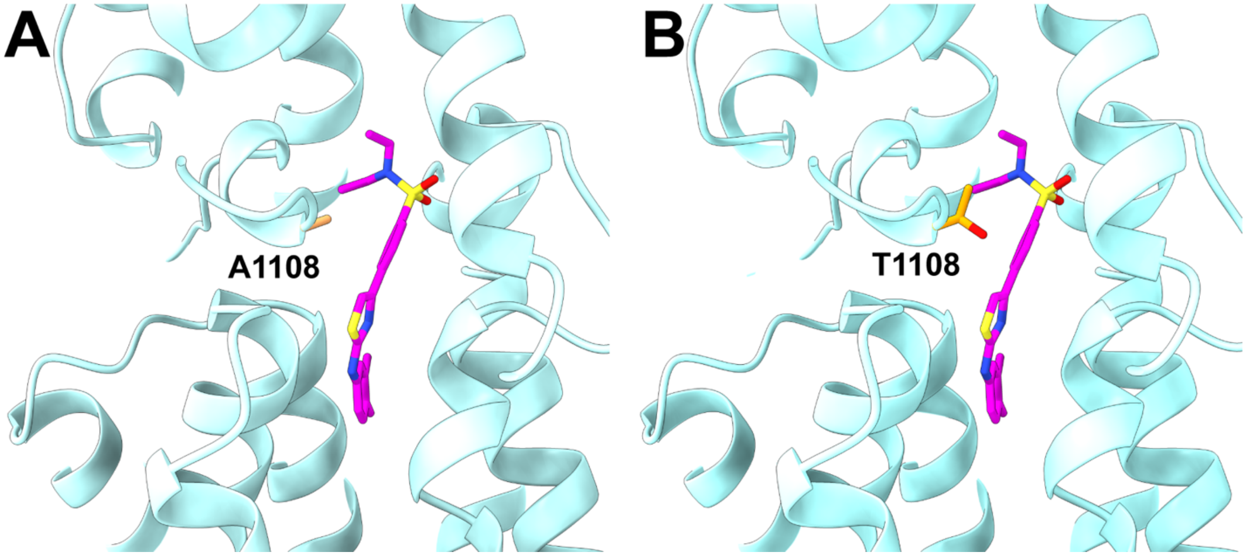

Fig. S9. Predicted potential steric hinderance of the A1108T mutant with the MMV009108 inhibitor. (A) Structure of the wild-type PfNCR1 transporter indicates that residue A1108 is ~3.6 Å away from bound MMV009108 (magenta). (B) Predicted structure of the A1108T mutant depicts that residue T1108 is ~2.1 Å away from bound MMV009108 (magenta), causing severe steric hindrance and restraining the binding of the MMV009108 inhibitor.

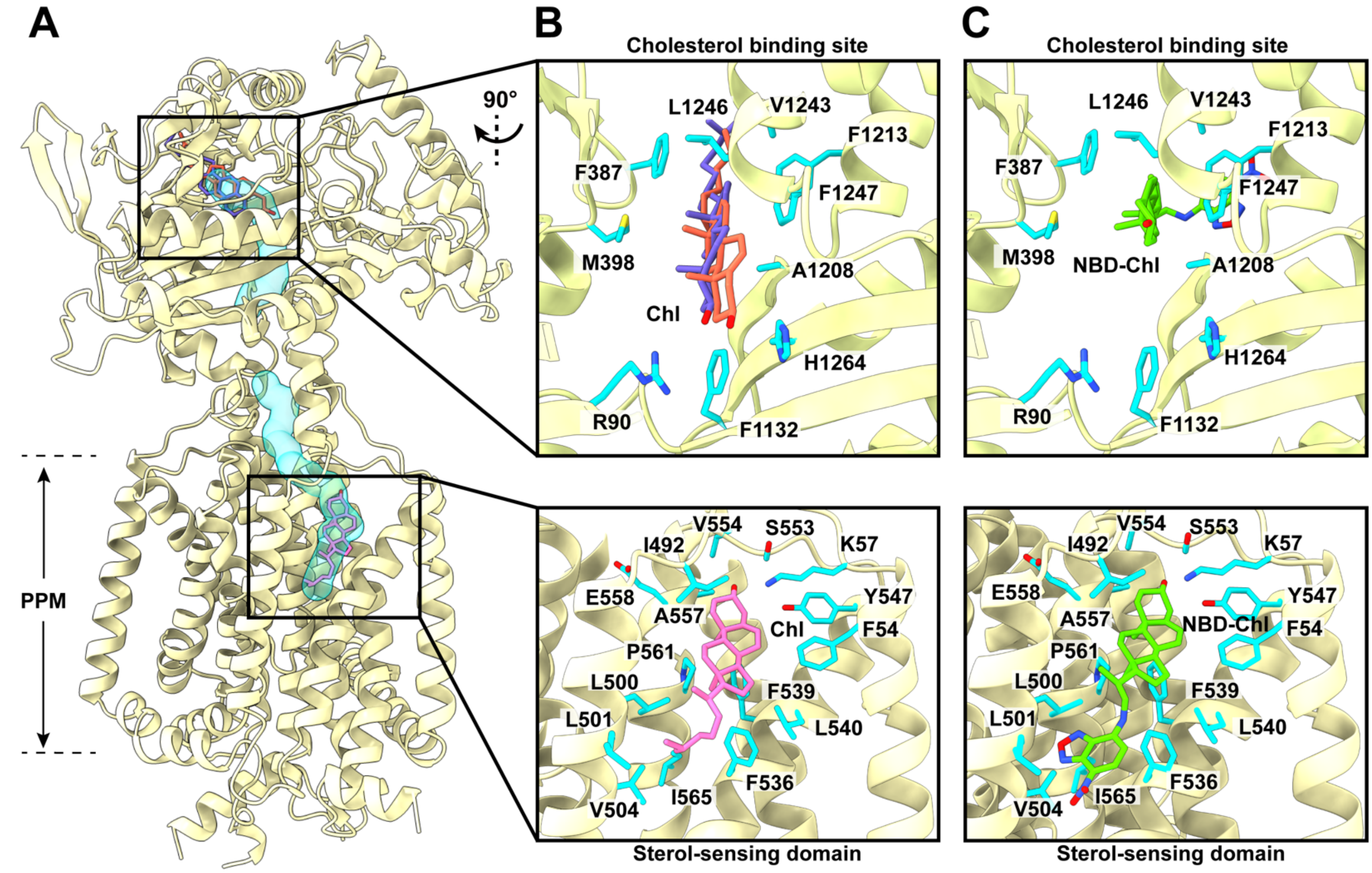

Fig. S10. Docking of cholesterol and NBD-cholesterol to the structure of PfNCR1. (A) The docking calculations indicate that there are two potent cholesterol (Chl)-binding sites located at the PV and PPM domains, respectively. Both docked Chl molecules are found within the tunnel (colored cyan) formed by the PfNCR1 transporter. (B) The docked Chl molecule at Chl-binding site of the PV domain is shown as orange sticks. The bound Chl ligand identified from our cryo-EM structure is also included and shown as slate sticks. Residues responsible for Chl binding are in cyan sticks. The second predicted Chl-binding site is located at the SSD of the PPM domain of PfNCR1. The docked Chl molecule at this binding site is shown as magenta sticks. Residues predicted to be responsible for Chl binding are in cyan sticks. (C) The docked NBD-Chl molecule at NBD-Chl-binding site of the PV domain is shown as green sticks. Residues responsible for NBD-Chl binding are in cyan sticks. The second predicted NBD-Chl-binding site is located at the SSD of the PPM domain of PfNCR1. The docked NBD-Chl molecule at this binding site is shown as green sticks. Residues predicted to be responsible for NBD-Chl binding are in cyan sticks.

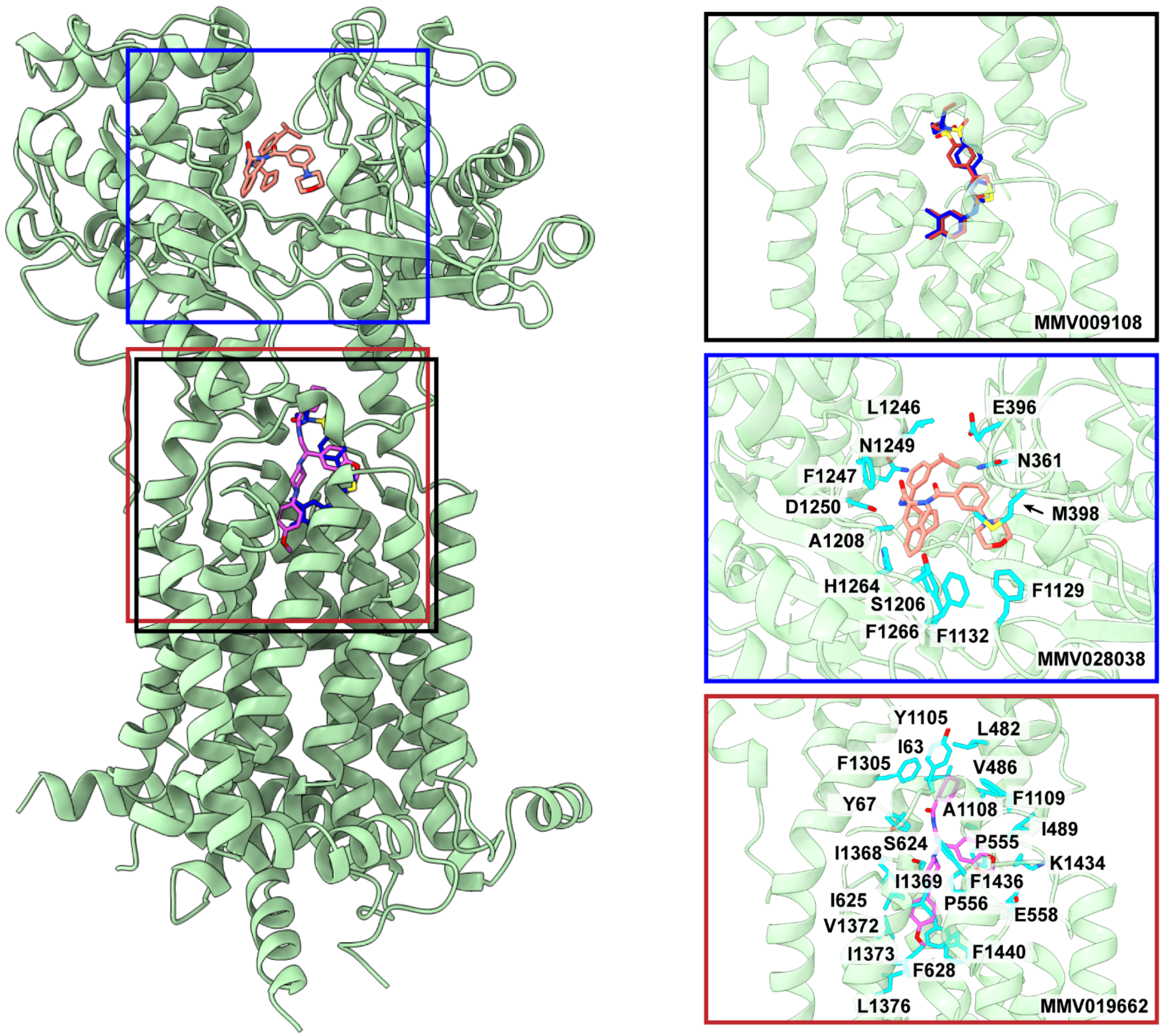

Fig. S11. Docking of inhibitors to the structure of PfNCR1. The docking calculations indicate that both MMV009108 (blue) and MMV019662 (magenta) are bound in the middle of the transporter and nearby the restriction site of the tunnel formed by PfNCR1, whereas MMV028038 (orange) is bound within the cleft between subdomains PV1 and PV2, and overlapped with the Chl binding site at the PV domain identified by cryo-EM. The upper insert panel depicts bound MMV009108 (red) identified by cryo-EM and docked MMV009108 (blue) predicted via docking. The middle insert panel indicates the predicted MMV028038 binding site. The bound MMV028038 molecule is colored orange, whereas residues within 4 Å of bound MMV028038 are in cyan sticks. The lower insert panel shows the predicted MMV019662 binding site. The bound MMV019662 molecule is colored magenta, whereas residues within 4 Å of bound MMV019662 are in cyan sticks.

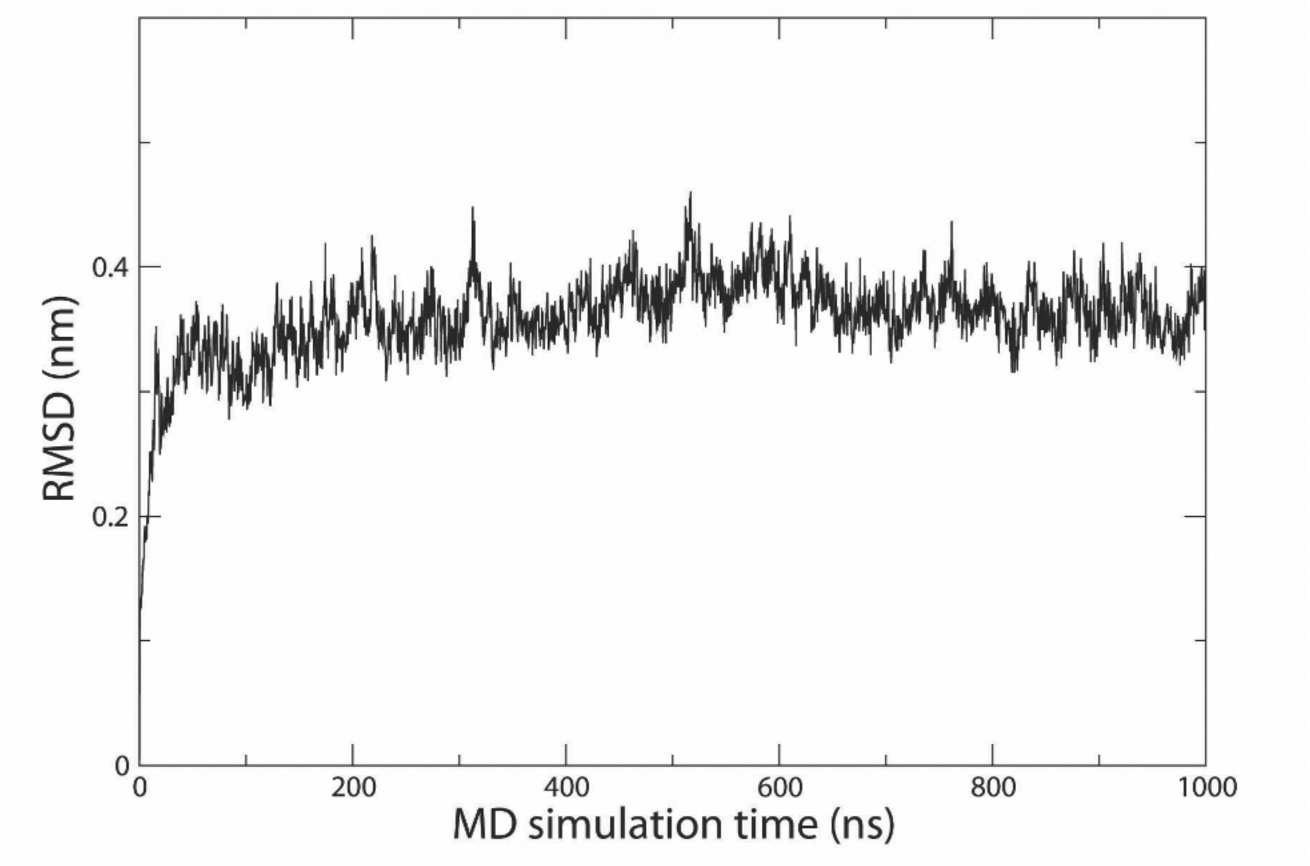

Fig. S12. Results of 1 □s Simulation. MD simulation results of the PfNCR1-I. The Cα atom RMSD (root mean square deviation) plot is based on the MD simulation trajectories (1 □s).

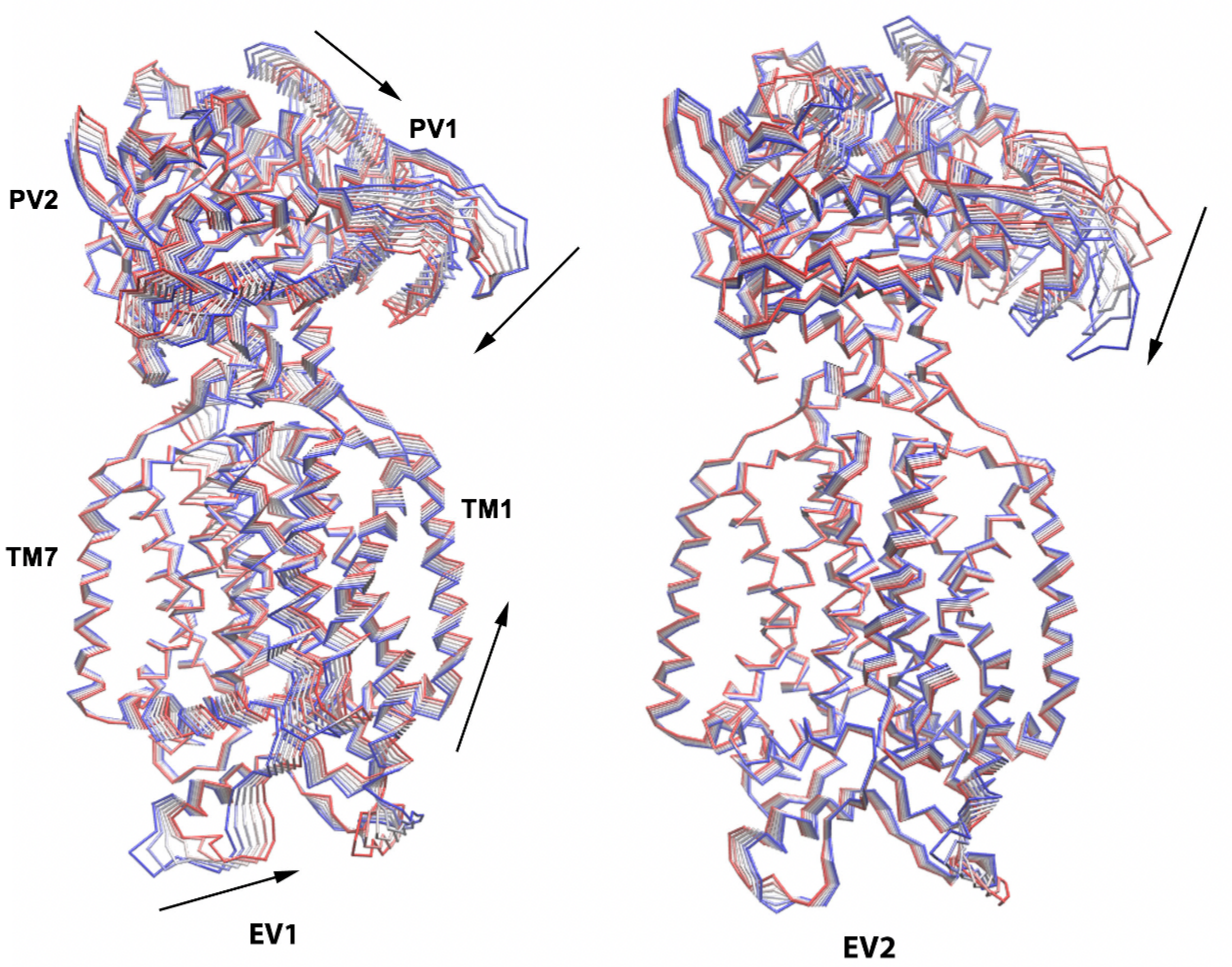

Fig. S13. The first and second eigenvectors of PfNCR1 from PCA. Black arrows show the rigid-body motions of PV and PPM domains. Six structures from each eigenvector were overlaid showing these rigid-body motions (blue to red).

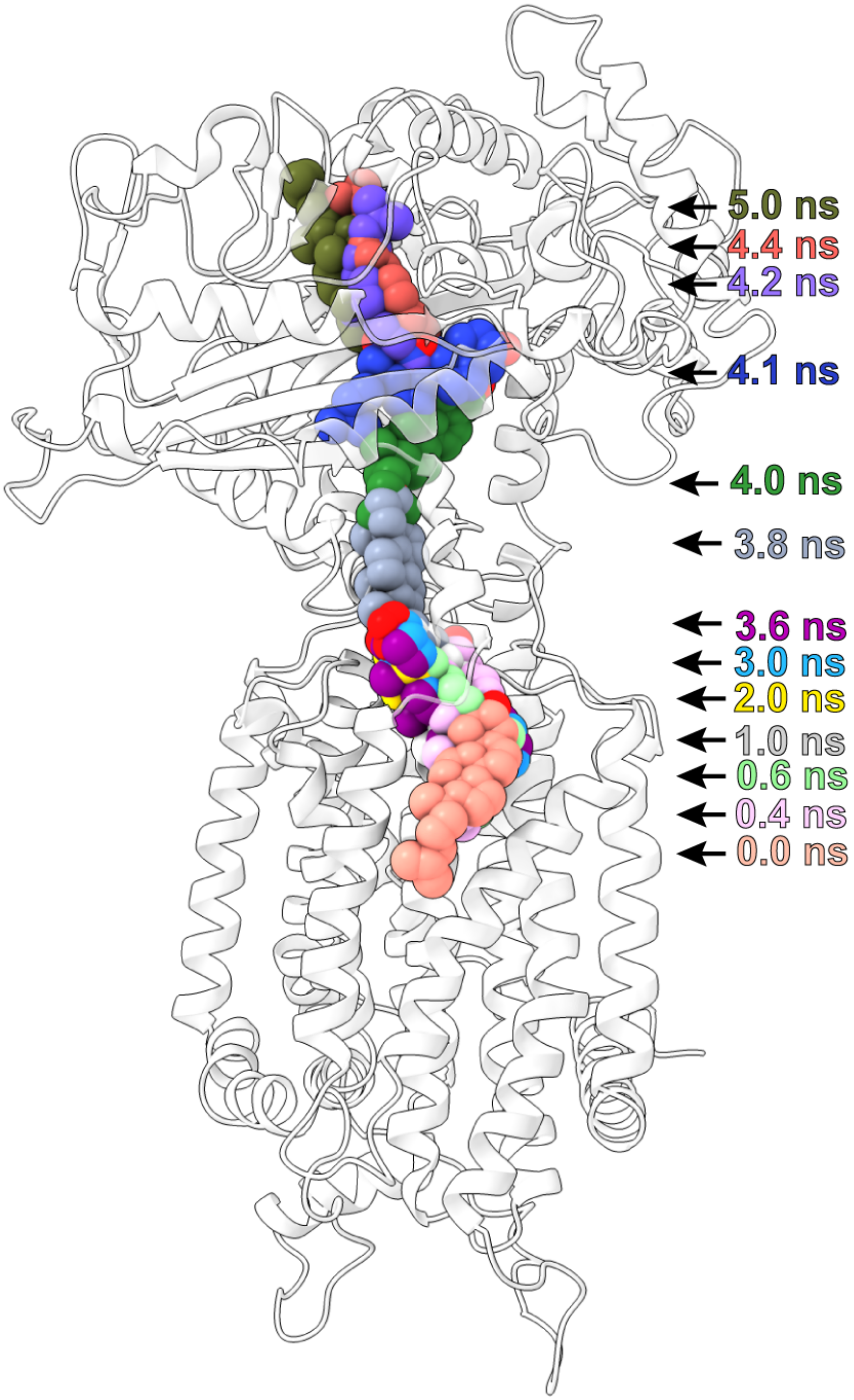

Fig. S14. Targeted MD simulations of the PfNCR1 transporter. The calculations depict snapshots (0, 0.4, 0.6, 1.0, 2.0, 3.0, 3.6, 3.8, 4.0, 4.1, 4.2, 4.4 and 5.0 ns) of Chl shuttling across the tunnel formed by PfNCR1.

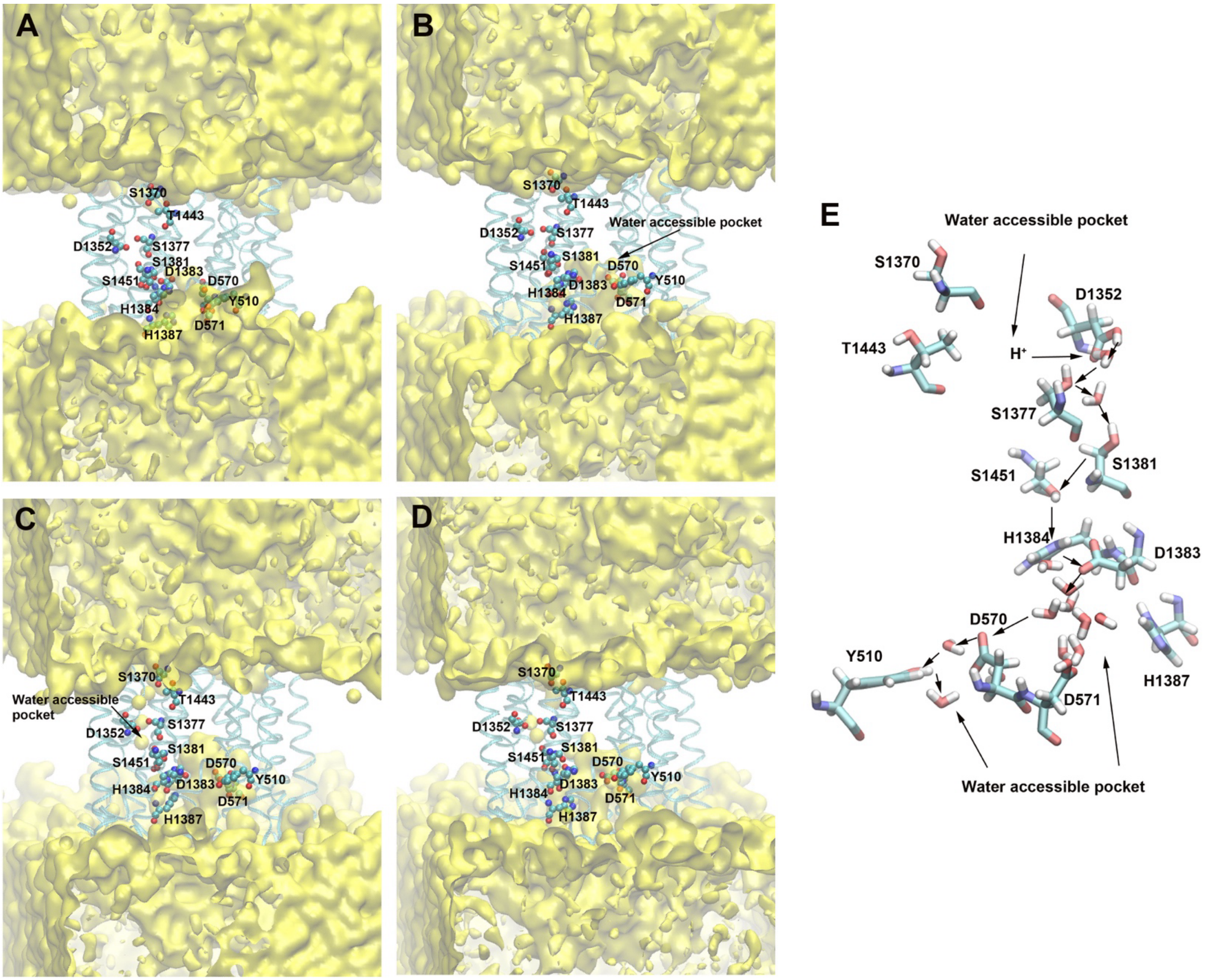

Fig S15. Putative proton transfer pathway of PfNCR1. This figure depicts snapshots of residues participating in the putative proton-relay network of PfNCR1 at (A) 0 ns, (B) 500 ns, (C) 593 ns and (D) 1000 ns. The simulations indicate that residues Y510, D570, D571, D1352, S1370, S1377, S1381, D1383, H1384, H1387, T1443 and S1451 form a proton-relay network to facilitate the transfer of proton across the membrane.

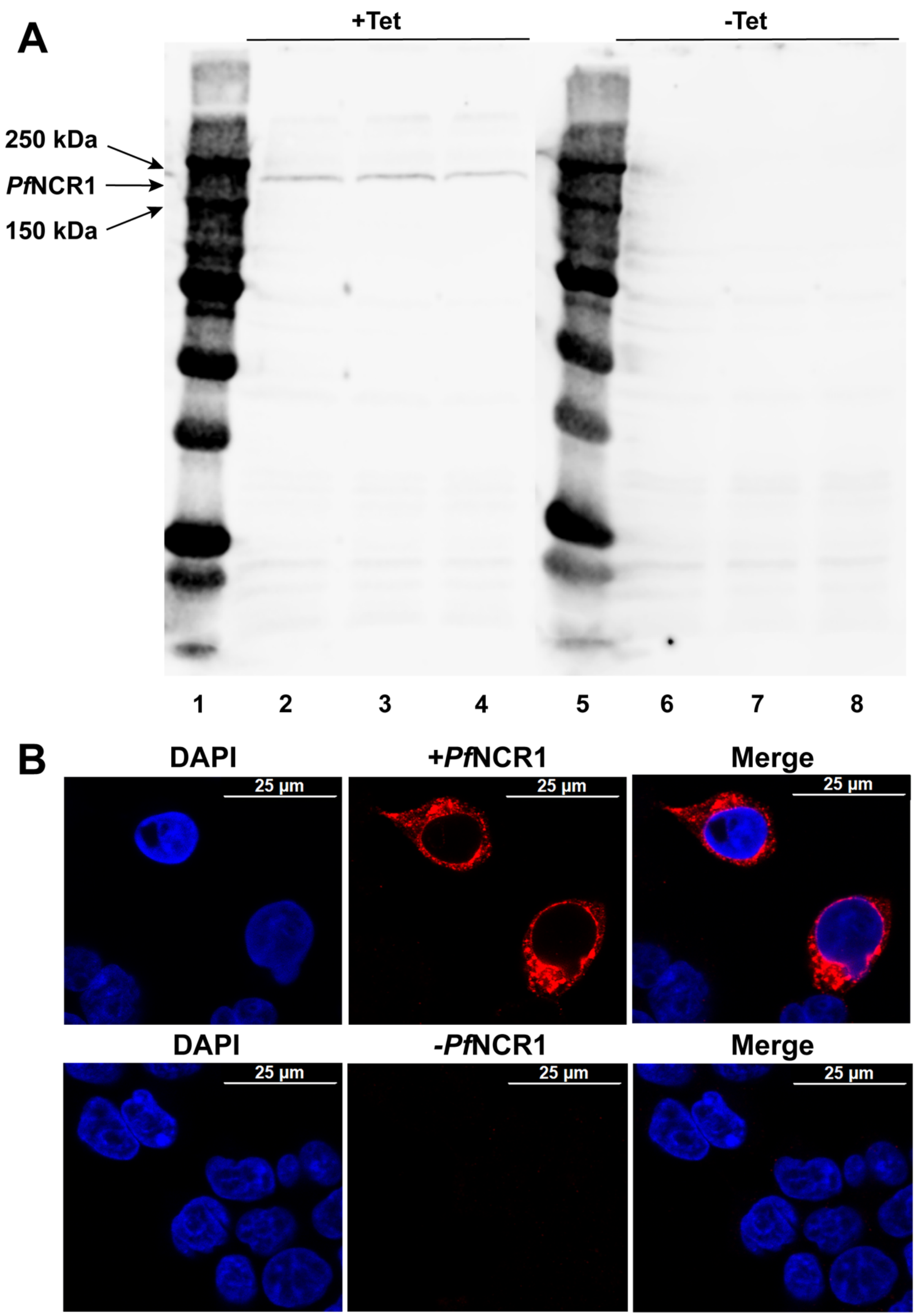

Fig. S16. Expression of the PfNCR1 transporter. (A) This is an immunobolt against PfNCR1 of membrane extracts from 50 [illegible]g of dry HEK293 cells expressing the PfNCR1 transporter after starvation (lanes 1 to 8 from right to left: marker, HEK293 cells expressing PfNCR1 (+ tet), HEK293 cells expressing PfNCR1 (+ tet), HEK293 cells expressing PfNCR1 (+ tet), marker, control HEK293 cells (- tet), control HEK293 cells (- tet) , control HEK293 cells (- tet)). (B) Representative confocal microscopy image showing PfNCR1 expressing HEK293 cells (top row) and non-PfNCR1 expressing HEK293 control cells (bottom row) stained for nuclei (blue) and PfNCR1 (red).

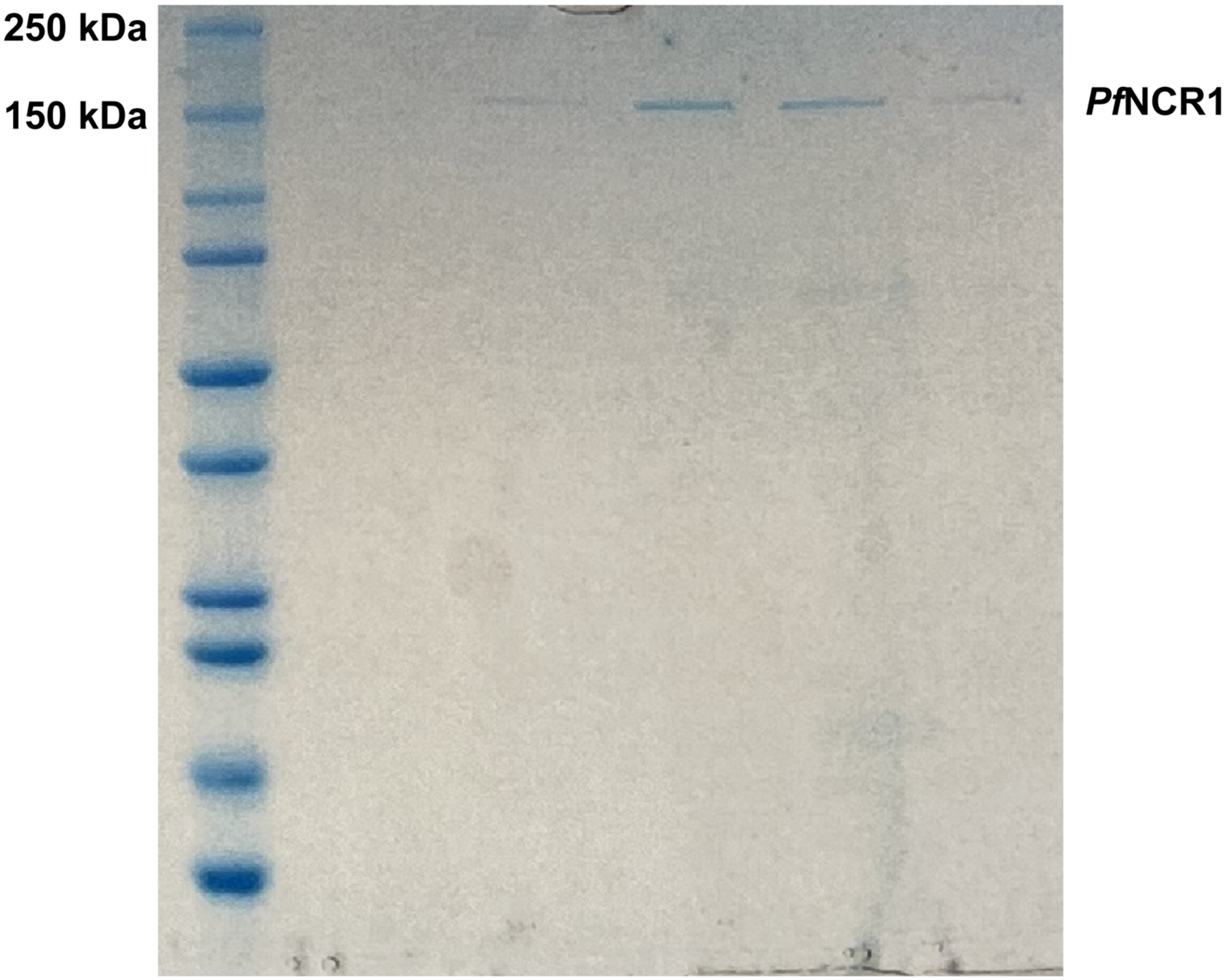

Fig. S17. SDS-PAGE of purified PfNCR1. The gel depicts that the purified PfNCR1 protein is >90% purity.

Table S1. PfNCR1 cryo-EM data collection and refinement statistics.

**Data collection**	**PfNCR1 in the absence of MMV009108**			**PfNCR1 in the presence of MMV009108**
Magnification	81,000	81,000	81,000	81,000
Voltage (kV)	300	300	300	300
Electron Microscope	Krios-GIF-K3	Krios-GIF-K3	Krios-GIF-K3	Krios-GIF-K3
Defocus (um)	-0.8 to -1.5	-0.8 to -1.5	-0.8 to -1.5	-0.8 to -1.5
Energy filter width (eV)	20	20	20	20
Pixel size (Å)	1.07 (0.535)	1.07 (0.535)	1.07 (0.535)	1.07 (0.535)
Total dose ($e^-/Å^2$)	35.9	35.7	33.0	40.5
Number of frames	38	38	40	45
Number of micrographs	1,268	3,042	2,618	4,065
Number of Initial particles	6,977,953			5,798,543

**Refinement**	**PfNCR1-I**	**PfNCR1-II**	**PfNCR1-MMV009108**
Number of total particles	63,097	56,123	82,204
GS-FSC Resolution (0.143, Å)	3.11	3.81	2.98
Model composition			
Chains	2	2	2
Protein residues	985	979	990
Ligand	3	2	4
r.m.s.d.			
Bond lengths (Å)	0.002	0.002	0.003
Bond angles (°)	0.460	0.480	0.445
**Validation**			
MolProbity score	1.51	1.89	1.30
Clash score	6.21	11.01	5.58
Ramachandran plot			
Favored (%)	98.87	98.55	98.37
Allowed (%)	1.13	1.45	1.63
Disallowed (%)	0.00	0.00	0.00
CC Mask	0.83	0.68	0.86
